# Bioinspired Injection Therapy for Spent LiFePO_4_ Batteries: A Non-Invasive Strategy for Capacity Regeneration and Longevity Enhancement

**DOI:** 10.1007/s40820-026-02091-1

**Published:** 2026-02-09

**Authors:** Peng Wang, Jian Wang, Longwei Bai, Na Li, Chuancong Zhou, Mingyang Chen, Jialiang Zhang, Zhenyue Xing, Zaowen Zhao, Wei Zhang, Xiaodong Shi

**Affiliations:** 1 Hebei Key Laboratory of Flexible Functional Materials, School of Materials Science and Engineering, Hebei University of Science and Technology, Shijiazhuang, 050018 People’s Republic of China; 2 State Key Laboratory of Tropic Ocean Engineering Materials and Materials Evaluation, School of Materials Science and Engineering, Hainan University, Haikou, 570228 People’s Republic of China; 3 State Key Laboratory of Advanced Metallurgy, University of Science and Technology Beijing, No. 30 Xueyuan Road, Haidian District, Beijing, 100083 People’s Republic of China; 4 Christopher Ingold Laboratory, Department of Chemistry, University College London, London, WC1H 0AJ UK; 5 College of Physics and Electronic Engineering, Nanyang Normal University, Nanyang, 473061 People’s Republic of China

**Keywords:** Spent LFP batteries, Direct regeneration, I_3_^−^/I^−^ redox couple, Dead lithium, Lithium replenishment

## Abstract

The widespread deployment of lithium iron phosphate (LiFePO_4_, LFP) batteries has intensified the imperative to address the disposal challenges associated with retired LFP batteries, given their rapidly growing volumes. However, existing regeneration techniques remain constrained by their inherent complexity, high energy demands, and limited scalability, posing significant barriers to achieving efficient and economically viable solutions. Herein, inspired by medical injection therapy, a novel, non-invasive strategy for direct capacity rejuvenation is proposed by injecting recovery reagents into spent LFP batteries, circumventing the need for disassembly. This innovative approach leverages the I_3_^−^/I^−^ redox couple to activate residual/dead lithium on the graphite anode and selectively re-engineer the solid electrolyte interphase (SEI), preserving its functional components while optimizing interfacial dynamics. The restored lithium from the anode serves as an intrinsic source to replenish lithium deficits and rectify Li–Fe antisite defects within the degraded LFP cathode. The resulting regenerated pouch cells demonstrate remarkable recovery of electrochemical capacity, accompanied by superior kinetics performance and significantly extended cycle life. This pioneering strategy not only delivers an energy-efficient and cost-effective pathway for LFP battery regeneration but also holds transformative potential to redefine sustainable practices in lithium-ion battery reuse, thereby advancing their practical applications and prolonging their service life.

**Supplementary Information:**

The online version contains supplementary material available at 10.1007/s40820-026-02091-1.

## Highlights

- An effective and facile injection strategy based on iodine redox chemistry is proposed to restore lost lithium component in spent LiFePO_4_ battery.
- In-situ spectroscopic characterization is conducted to unveil the underlying redox mechanism of iodine-mediated lithium reactivation, elucidating its impact on both the cathode and anode.
- Regenerated LiFePO_4_ cells exhibit ~ 7% capacity recovery, fast charge transfer behavior, and extended cycle life beyond 300 cycles at 1C, outperforming conventional direct recycling methods while maintaining superior cyclic stability.

**Supplementary Information:**

The online version contains supplementary material available at 10.1007/s40820-026-02091-1.

## Graphical abstract

**Figure:**
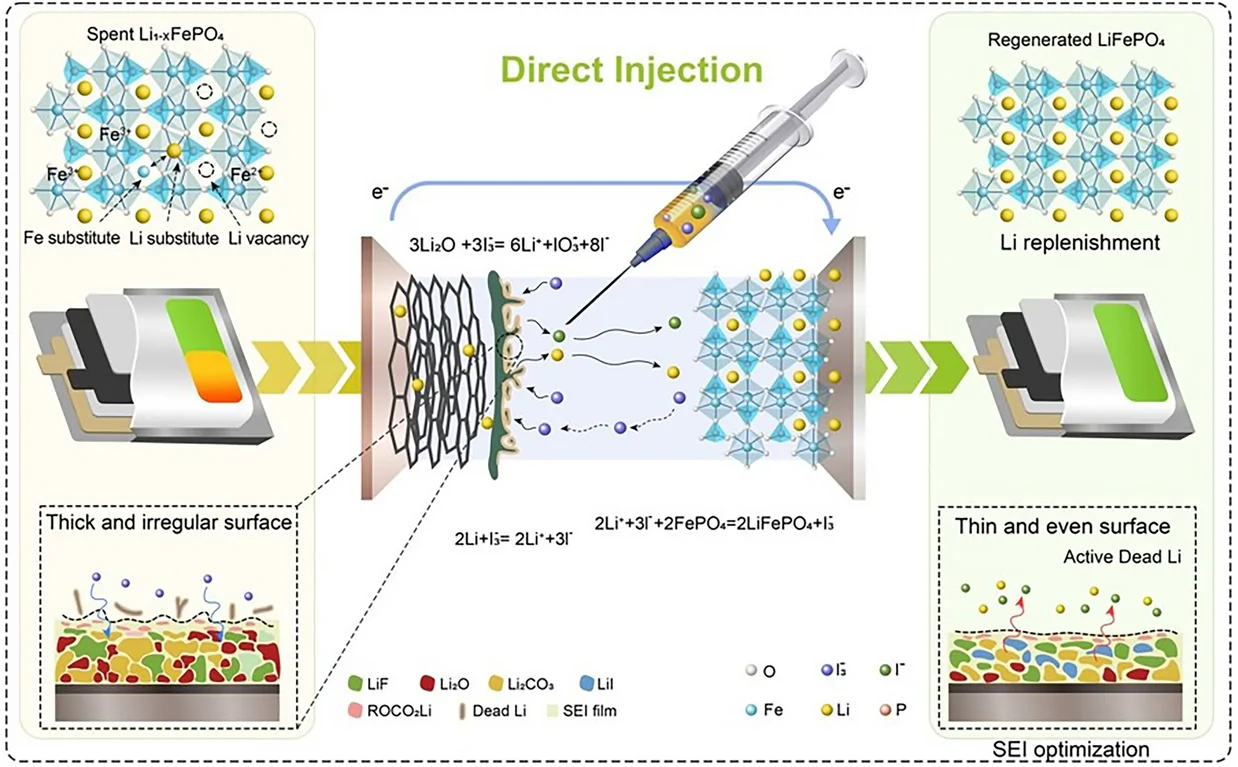

## Introduction

The rapidly expanding market share of olivine lithium iron phosphate (LiFePO_4_, LFP) batteries is driven by their low cost, long cycle life, and excellent safety profile, especially within the realms of electric vehicles (EVs) and grid energy storage applications [1–3]. Furthermore, the global sales volume of EVs and hybrid EVs powered by LFP batteries has exceeded 1,000,000 now, accounting for over one in three of the entire lithium-ion batteries market [4, 5]. Therefore, the impending retirement of vast quantities of spent LFP batteries underscores the urgent need for efficient and sustainable recycling methods that minimize resource and energy consumption while mitigating environmental impact [6].

As illustrated in Fig. S1, conventional lithium-ion battery recycling involves disassembling spent batteries and separating their components. These processes predominantly focus on recovering materials, particularly cathodes, through hydro- or pyro-metallurgical refining into raw materials, which are subsequently used to resynthesize electrode materials (Type A in Fig. S1) [7–9]. While effective for high-value materials, such as those containing nickel and cobalt, these approaches are economically unfeasible for spent LFP batteries, which lack high-value metals and thus fail to offset the costs associated with hydro- or pyro-metallurgical recycling [10]. Recently, direct recycling has emerged as a viable alternative with significant potential, aiming to reactivate electrode materials without reprocessing them into raw materials (Type B in Fig. S1) [11, 12]. For instance, Cheng et al. explored a direct recycling strategy by simple solid–liquid reaction, manifesting huge economic advantages over traditional recycling techniques [13]. Nevertheless, these techniques typically require additional steps, including battery disassembly and reassembly, which entail substantial time, energy, and manufacturing costs. Thus, there is an urgent need for streamlined, cost-effective solutions to exploit the residual value of degraded batteries while minimizing process complexity.

By and large, the capacity degradation of lithium-ion batteries over prolonged usage arises principally from two factors: (1) loss of active electrode materials ascribed to structural disorder, particle cracking, and loss of electrical contact; (2) lithium-ion loss stemming from surface film formation (*e.g.*, SEI growth), decomposition reactions, and the formation of inactive lithium (*e.g.*, dead lithium) [14–16]. Recent studies have highlighted dead lithium, rather than SEI compounds, as the dominant contributor to capacity fade [17–19]. This phenomenon impairs both electrodes, as the inaccessibility of lithium ions precludes their reinsertion into the active structure [20]. However, the ideal solution of capacity decay by active lithium loss should not impact the structural integrity of the electrodes [21]. For LFP cathodes, whose olivine structure remains stable over extended cycling, performance degradation is primarily attributed to lithium deficiency [22]. Additionally, irreversible lithium extraction induces Li/Fe cation site exchange, resulting in Li–Fe antisite defects that impede Li^+^ diffusion pathways [23]. Encouragingly, lithium replenishment and Fe(III) reduction have proven effective in mitigating these defects [24]. Guo et al. reported a straightforward method in-situ regenerating the delithiated LFP through a graphite prelithiation strategy, which delivers a novel route to regenerated LFP cells from graphite anode side but requires reassembly process [25]. Ogihara et al. reported a direct capacity regeneration technique through the injection of Li-Naph-based recovery reagent, which is capable of selectively offering both electrons and carrier Li ions to the cathode and resulting in capacity recovery without degradation with cycles [26]. Typically, substantial amounts of active lithium are typically sequestered in the graphite anode in the form of SEI, lithium deposits, and dead lithium [27–29], which are electrochemically inactive. Therefore, the reutilization of residual lithium from spent anode provides a promising opportunity for directly regenerating spent LFP cathode, which can preserve the structural integrity and capitalize the straightforward regeneration mechanism of LFP.

**Fig. 1:**
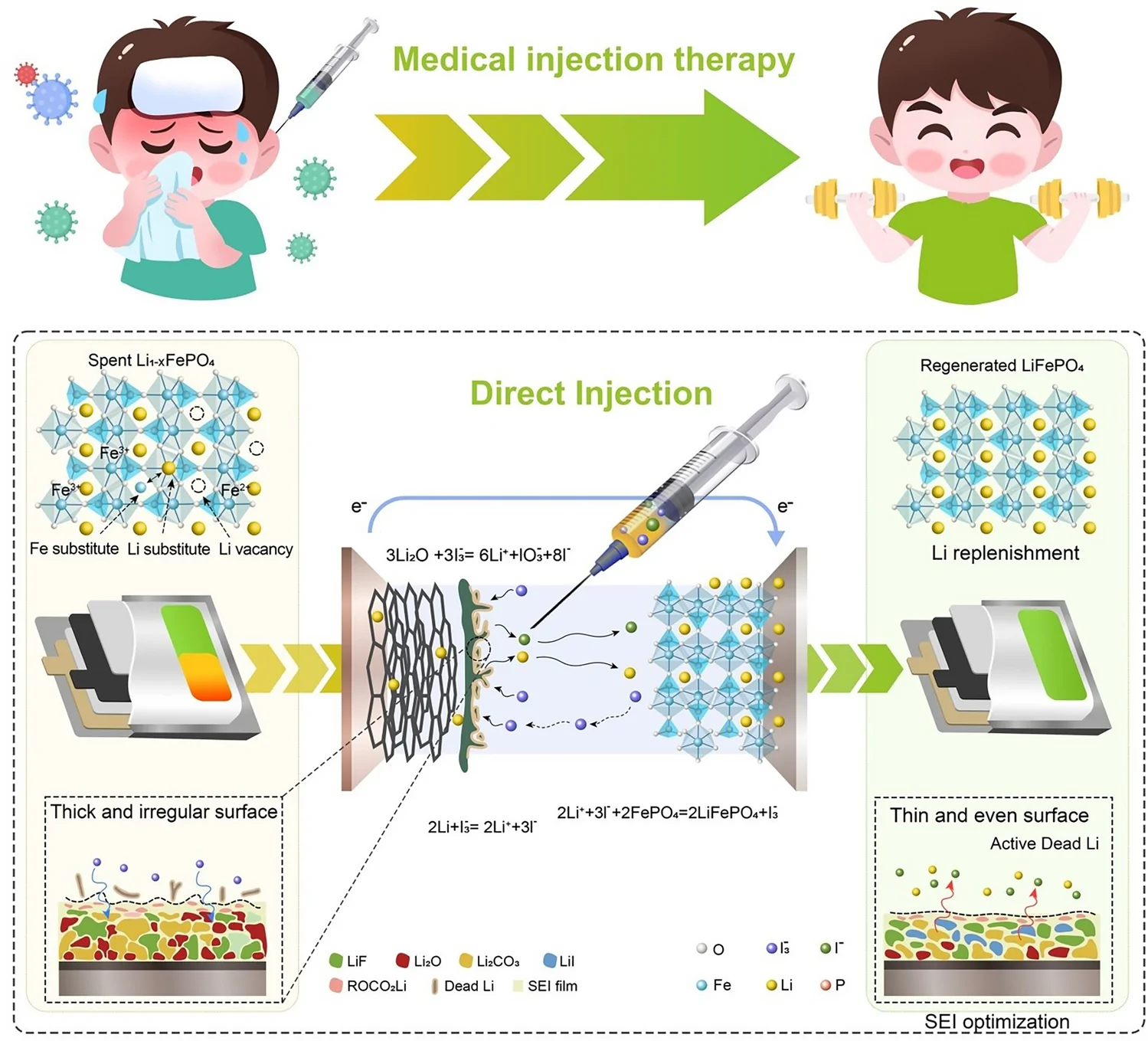
Schematic illustration of the action mechanism of the I_3_^−^/I^−^ redox couple strategy for the direct regeneration of spent LiFePO_4_/graphite(LFP/Gra) pouch cell

Herein, inspired by the principles of medical treatment through injection therapy, we propose a novel strategy for capacity restoration by injecting recovery reagents into spent LFP cells (Fig. 1). In the medical field, injection therapy is widely used to deliver targeted treatments directly into affected areas, facilitating rapid recovery and restoring function without invasive procedures. Analogously, our method mirrors this approach by introducing an I_3_^−^/I^−^ redox couple as a “therapeutic agent” to rejuvenate degraded lithium-ion cells in situ, without the need for disassembly (Type C in Fig. S1). Just as injection therapy aids in healing damaged tissues or restoring biological balance, our redox-based injection strategy reactivates dead lithium as the primary lithium source, directly replenishing lithium losses and revitalizing electrochemical performance [30]. The I_3_^−^/I^−^ redox couple plays a crucial role in this process, effectively converting inactive lithium deposits on the graphite anode surface into mobile Li^+^ ions. These liberated Li^+^ travel back to the delithiated cathode, where they reduce Fe(III) to Fe(II) and eliminate Li–Fe antisite defects, akin to how targeted medication restores cellular function. Simultaneously, the injected reagents optimize the SEI on the graphite anode, reinforcing interfacial stability much like how therapeutic injections strengthen biological structures. Through this multifunctional and non-invasive approach, we successfully regenerated spent pouch cells, achieving a remarkable capacity recovery of approximately 7% and superior cyclic stability. The rejuvenated cells sustained over 300 cycles at 1C, demonstrating a significantly extended lifespan. This bioinspired, economical, and effective strategy redefines spent cell regeneration by providing a practical and scalable alternative to conventional recycling processes. By leveraging a targeted and restorative methodology akin to medical injection therapy, our approach maximizes the potential for industrial adoption and promotes a sustainable battery economy, ultimately contributing to a circular energy landscape [31].

## Experimental Section

### Preparation of Recovery Reagent

Iodine (I_2_, AR, 99.9%), lithium iodide (LiI, AR, 99.9%), ethylene carbonate (EC, AR, 99.9%), diethyl carbonate (DEC, AR, 99.9%), and dimethyl carbonate (DMC, AR, 99.9%) were purchased from Aladdin Chem. Co., Ltd. A commercial ester-based electrolyte (1 M LiPF_6_ in EC/DEC/DMC (1:1:1 in volume) was obtained from Dodochem Technology. Recovery regent in this work was prepared by adding 0.01 M I_2_ into the commercial electrolyte and then stirring for 1 h. After introducing iodine species, the color of recovery regent changed to reddish-brown. Additionally, LiI was dissolved into DMC solvent and regarded as a control solution for further investigation. All the raw material samples mentioned above were directly used in the experiment without undergoing any further purification or purification processes.

### Preparation of the Spent Electrode Materials

Commercial LiFePO_4_/graphite (LFP/Gra) pouch cells of Great Power Battery Co., Ltd. were cycled within 2.5–4.2 V at 1C (current density ~ 200 mA g^−1^) and 25 °C until the capacity retention ratio decreases to ~ 80%. Then, the degraded pouch cells were disassembled in an Ar-filled glovebox, and the spent LFP cathode (abbreviated as S-LFP) and spent graphite anode (abbreviated as S-Gra) were obtained after fully washed with DMC solvent to remove the remaining electrolyte in glovebox and stored at 80 °C for 12 h in a vacuum oven.

### Preparation of the Regenerated Electrode Materials

The degraded LFP/Gra pouch cells were pricked a hole from the bottom in an Ar-filled glovebox, and 0.2 mL of I_2_-containing recovery reagent was injected by a needle tube. The pinhole was further sealed with an electrolyte-resistant sealing tape (lithium battery termination tape, Commodity Code: 99007200, Shenzhen Kejing Zhida Technology Co., Ltd.) in Ar-filled glovebox and rested for 24 h. Subsequently, the injected pouch cells were cycled within 2.5–4.2 V at 1C for 10/20 cycles for the comprehensive electrode regeneration. Then, the targeted pouch cells were manually disassembled, and the regenerated LFP cathode (abbreviated as R-LFP) and the regenerated graphite anode (abbreviated as R-Gra) were washed with DMC solvent to remove residual electrolyte in glovebox and stored at 80 °C for 12 h in a vacuum oven.

### Material Characterizations

The phase and crystal structure of the obtained electrode materials were investigated via X-ray diffractometer (Bruker D8 Advance) with Cu Kα radiation was applied to analyze the phase structure and anti-site defects within 10°–90° (Scan rate: 2° min^−1^). The microstructures of electrode materials were observed by transmission electron microscope (TEM, FEI Talos F200S, USA). Scanning electron microscope (SEM, Hitachi SU8010), combined with energy-dispersive X-ray spectroscopy (EDS), was performed to examine the microscopic morphology and elemental distribution of the electrodes. Raman spectra were detected on a Thermo Fisher DXR spectrometer. X-ray photoelectron spectroscopy (XPS, Thermo Fisher ESCALAB Xi +) with Al Kα radiation was conducted to analyze the elemental composition and bonding states based on the calibration of standard C 1*s* peak at 284.8 eV. Accurate elemental contents were detected by inductively coupled plasma optical emission spectrometry (ICP-OES, PerkinElmer Avio500). Ultraviolet spectrophotometry (UV–Vis, TU-1901, Beijing PuXi General Instrument Co., Ltd.) was employed to monitor signal changes of I^−^ and I_3_^−^ species in the electrolyte.

### Electrochemical Measurements

Cyclic voltammetry (CV) curves of pouch cells were conducted within the voltage range of 2.5–4.2 V at 0.1 mV s^−1^, and electrochemical impedance spectroscopy (EIS) of pouch cells was measured within 10 mHz–100 kHz. Both of them were implemented on the electrochemical workstation (Shanghai Chenhua Instrument, CHI660E). To conduct the in situ UV–Vis spectroscopy, the S-LFP and S-Gra electrodes were cut into rectangular shapes (0.5 cm × 1 cm) and sealed in a homemade-cuvette cells in Ar-filled glovebox, incorporating with 3 mL recovery reagent, which was galvanostatically cycled at 0.1C on CHI660E electrochemical workstation. The corresponding UV–Vis spectra were collected at fixed intervals during cycling process within the wavelength range of 190–500 nm.

### Calculation Details

All density functional theory (DFT) calculations were detected utilizing Gaussian 09 [32]. The different conformers were optimized by the B3LYP [33]/def2tzvpp [34, 35] functional. To confirm the stability of the optimized structures, harmonic vibrational frequencies were calculated, ensuring all frequencies were positive. The overall Gibbs free energy change (∆G) of a reaction was determined as the difference in energy between the products and reactants, expressed as ∆G = G_products_—G_reactants_. Quantum chemical calculations were employed utilizing the Gaussian 16 program package [32]. The frontier molecular orbital energies were calculated by the B3LYP/def2tzvpp functional to optimize the molecular structure for a stable configuration. Single-point energy calculations were then employed utilizing the same functional to calculate the molecular orbital data.

## Results and Discussion

### Surface Transformation of Graphite Anode During the Regeneration Process

The working mechanism of the regeneration strategy based on the I_3_^−^/I^−^ redox couple for the spent LiFePO_4_/graphite (LFP/Gra) pouch cells (Fig. 1) can be mainly divided into three steps: (1) The I_3_⁻ species in the recovery reagent reacts with the inactive lithium on the spent graphite anode and converts dead lithium component (Li/Li_2_O) into the electrochemically active Li^+^ ions (Eqs. 1 and 2) [36, 37]; (2) these Li⁺ ions are shuttled back to the spent LFP cathode, and spontaneously re-intercalated into the lattice structure of devitalized FePO_4_, which effectively reconstructs the LiFePO_4_ phase and mitigates the Li–Fe antisite defects in the regenerated LFP cathode (Eq. 3) [38]; (3) the participation of I_3_^−^/I^−^ redox couple in the LFP/Gra pouch cells can continuously optimize the SEI components of graphite anode, regulate the lithium-ion deposition behavior, avoid the formation of dead lithium, and ensure relatively stable cyclic behavior [39, 40]. Generally, IO_3_⁻ ion is generated via the reaction between I_3_⁻ and Li_2_O, which also is a stable and fully oxidized iodine species. Within the operational voltage of LFP/Gra pouch cells (2.5–4.2 V), IO_3_⁻ is not expected to undergo further reaction. Therefore, it likely remains as an electrochemically inert component dissolved in the electrolyte. Meanwhile, the generated IO_3_⁻ amount is stoichiometrically linked to the consumption of Li_2_O, which only originates from the initial SEI layer of S-Gra anode. Based on this, we can conclude that the IO_3_⁻ species will not accumulate in the regenerated cells during the cycle process, and the trace amount of IO_3_⁻ species initially generated by the reaction between I_3_⁻ and Li_2_O also has a negligible impact on the battery performance.

1 $$2{\text{Li }} + {\text{ I}}_{3}^{ - } = \, 2{\mathrm{Li}}^{ + } + \, 3{\mathrm{I}}^{ - }$$

2 $$3{\mathrm{Li}}_{2} {\text{O }} + \, 3{\mathrm{I}}_{3}^{ - } = \, 6{\mathrm{Li}}^{ + } + \, 8{\mathrm{I}}^{ - } + {\text{ IO}}_{3}^{-}$$

3 $$3{\mathrm{I}}^{ - } + \, 2{\mathrm{FePO}}_{4} + \, 2{\mathrm{Li}}^{ + } = {\text{ I}}_{3}^{ - } + \, 2{\mathrm{LiFePO}}_{4}$$

**Fig. 2:**
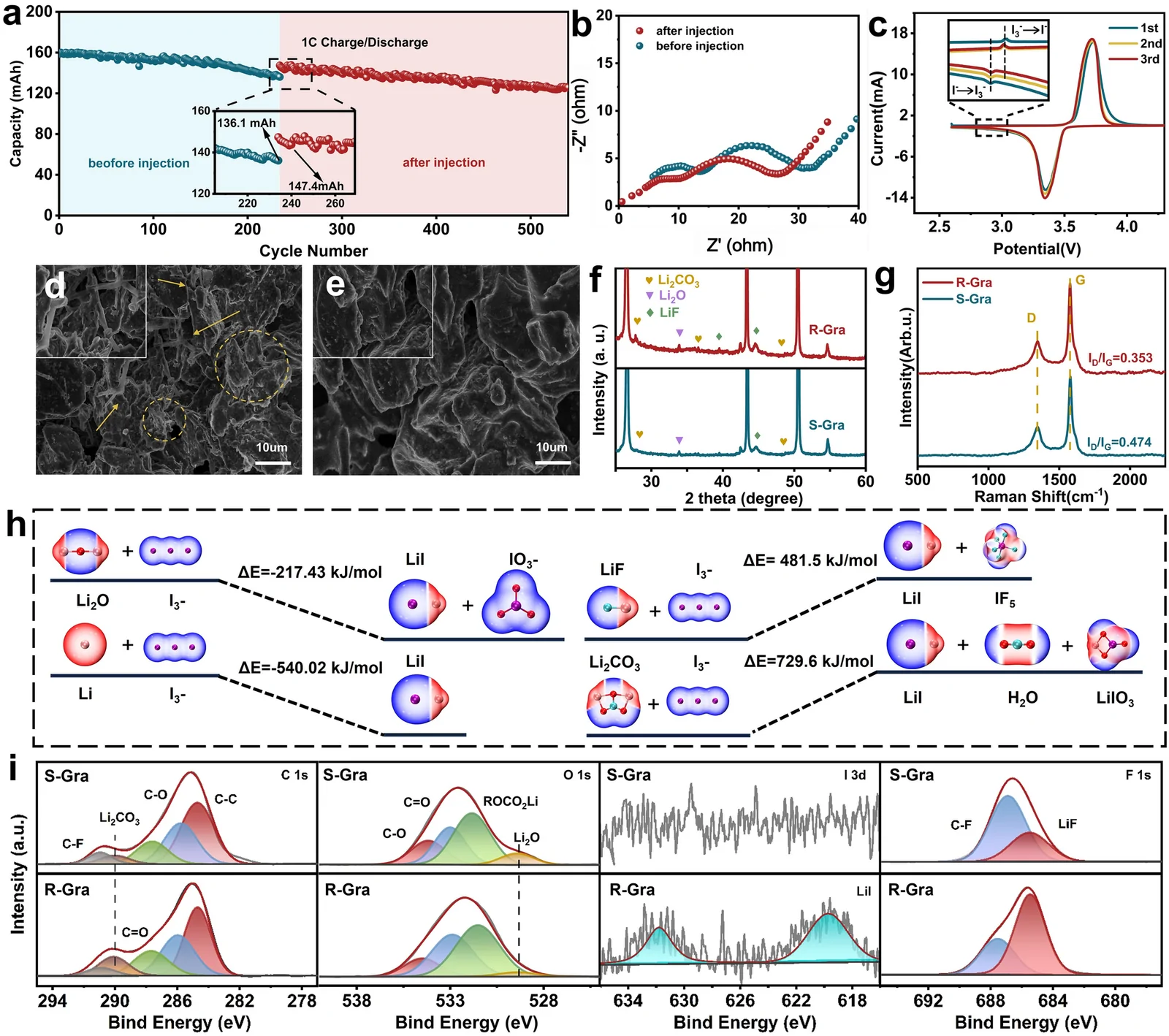
**a** Cycling performance of pouch cells before and after injection at 1C. **b** EIS spectra of pouch cells before and after injection. **c** CV curves of spent pouch cells in the recovery reagent within 2.5–4.2 V at 0.1 mV s^−1^. SEM images of **d** S-Gra and **e** R-Gra anodes. **f** XRD patterns and **g** Raman spectra of S-Gra and R-Gra anodes. **h** Reaction energy (∆E) analysis for the interaction between I_3_^−^ species and Li/Li_2_O/LiF/Li_2_CO_3_ components; **i** High-resolution C 1*s*, O 1* s*, I 3*d*, and F 1*s* spectra of S-Gra and R-Gra anodes

Capacity-recovery study was conducted on the spent LiFePO_4_/graphite (LFP/Gra) pouch cells (Fig. S2). To evaluate the optimal dosage of I_2_-containing recovery reagent, the cycling performances of spent LFP/Gra pouch cells in I_2_-containing electrolyte with different I_2_ molar concentrations were investigated. As compared in Fig. S3, the reversible capacity increases with the increase in I_2_ molar concentration within 0.01 M. When the molar concentration exceeds 0.01 M, it begins to show a downward trend again, demonstrating the suitable I_2_ molar concentration was 0.01 M. After determining the optimal molar concentration in the recovery reagent, its optimal injection volume was further studied through the cyclic stability test of the pouch cells. As clearly presented in Fig. S4, the spent LFP/Gra pouch cells injected with 0.2 mL recovery reagent exhibit the highest capacity and the best cyclic stability. Thus, the optimal dosage of I_2_-containing recovery reagent was confirmed as 0.2 mL with 0.01 M I_2_ molar concentration. Figure 2a vividly contrasts the capacity difference of spent pouch cells before and after the injection of recovery reagent. In details, the pouch cells deliver high initial capacity of 160 mAh and only stabilize at 136.1 mAh after 234 cycles at 1C, corresponding to poor capacity retention ratio of 85.1%, which may be mainly attributed to the continuous accumulation of dead lithium on the surface of graphite anode. After injecting I_2_-containing recovery reagent, the corresponding capacity immediately recovers from 136.1 to147.4 mAh and remains 126.6 mAh after extra 316 cycles at 1C with high-capacity retention ratio of 85.9% [36]. For the fresh LFP pouch cells without the injection treatment of I_2_-containing recovery reagent, the remaining capacities are 136.85 and 116.54 mAh after 231 and 550 cycles, respectively (Fig. S5), while the calculated capacity retention ratio is only 72.8%. Table S1 systematically compares the cyclic stability of LFP pouch cells regenerated by different methods, and the high-capacity retention ratio of injection recovery strategy demonstrate this non-invasive restorative procedure contributes to fully activate and utilize the lithium resource that in inert/inactive state. These results confirm the feasibility of I_3_^−^/I^−^ redox couple in recovering the decayed reversible capacity during cycling process. Electrochemical impedance spectroscopy (EIS) results reveal the smaller interfacial resistances of pouch cells after injecting the recovery reagent (Fig. 2b), implying the fast charge transfer behavior driven by the I_3_^−^/I^−^ redox couple [41].

Characteristic redox peaks of I_3_^−^/I^−^ couple can be only detected near 2.9 V in the cyclic voltammetry (CV) curves of spent LFP/Gra cells in I_2_-containing electrolyte (Fig. 2c), but not appears in I_2_-free electrolyte (Fig. S6). This oxidation potential of I_3_^−^/I^−^ couple is below the operating voltage (> 3.0 V) of most cathode materials, which is beneficial for the nondestructive repair without affecting the normal operation of the battery [42]. Scanning electron microscopy (SEM) was conducted to reveal the morphological differences between S-Gra and R-Gra anodes. As presented in Fig. 2d, the rough and disordered surface of S-Gra anode is associated with the formation dead lithium, irregular SEI accumulation, and inhomogeneous Li deposition. In contrast, R-Gra anode exhibits a flat and clean surface, which can be ascribed to the efficient elimination of dead lithium and irregular SEI components by the I_3_^−^/I^−^ redox couple (Fig. 2e). X-ray diffraction (XRD) results further validate the changes of surface material components on S-Gra and R-Gra anodes. The characteristic diffraction peaks corresponding to the inorganic components, such as LiF, Li_2_O, and Li_2_CO_3_, can be clearly detected on both S-Gra and R-Gra anodes (Figs. 2f and S7) [43], while the qualitative content of Li_2_CO_3_ on S-Gra anode is higher than that on R-Gra anode, confirming its generation and accumulation after injection treatment, which is an inorganic component of SEI layer. Additionally, the disordered and graphitized degree of graphite anode is characterized by Raman spectroscopy. Compared with the bare graphite anode (B-Gra), the peak intensity ratio (I_D_/I_G_) of S-Gra remarkably escalates from 0.26 to 0.474 (Figs. 2g and S8), suggesting severe structural degradation and high amorphous degree after long-term cycling.

After the injection treatment, the I_D_/I_G_ ratio of R-Gra is calculated as 0.353, within the corresponding values of B-Gra and R-Gra, demonstrating the reduced disorder degree and the recovered graphitization degree. DFT calculations affirm the chemical reaction between I_3_^−^ species and Li_2_O or Li is energetically favorable (ΔE < 0 kJ mol^−1^) but unfavorable for the reaction between I_3_^−^ and LiF or Li_2_CO_3_ (Fig. 2h, ΔE > 0 kJ mol^−1^). This result suggests that I_3_^−^ species can selectively scavenge the Li_2_O/dead lithium components and preserve the LiF/Li_2_CO_3_ components that beneficial for the robust and stable SEI layer. Furthermore, the surface chemistry of S-Gra and R-Gra anodes was analyzed to investigate the composition changes in the SEI layer. High-resolution C 1*s* spectra for S-Gra and R-Gra show five distinct binding peaks at 284.75, 286.01, 287.85, 289.95, and 290.90 eV, corresponding to C–C/C–H, C–O, C=O, Li_2_CO_3_, and C–F groups, respectively [44, 45]. The C–C/C–H, C–O, and C–O peaks are attributed to the metastable organic components, originating from the decomposition of the ester-based solvents (Fig. 2i). Among these components, Li_2_CO_3_, as an exceedingly stable and essential SEI component [46], exhibits slightly content increase on the surface of R-Gra, indicating its formation and growth during the battery degradation process. This result is consistent with the XRD patterns in Fig. 2f. For high-resolution O 1*s* spectra, the characteristic peaks at 529.35, 531.50, 532.90, and 534.25 eV are observed, corresponding to Li_2_O, ROCO_2_Li, C=O, and C–O groups, respectively [47]. Compared to S-Gra, the proportion of Li_2_O significantly declines after injection treatment, indicating that Li_2_O is effectively reduced by I_3_^−^ additive [48], which contributes to activate dead lithium and reconstruct the components of SEI layer. For the high-resolution F 1 s spectra, the C-F peak at 687.95 eV dominates the main position before injection treatment, accompanying with a weaker LiF signal at 685.50 eV, confirm the fluorine element in the SEI layer of S-Gra primarily exists as organic fluorides, which always have poor ionic diffusion capability [49]. After injection treatment, a higher proportion of LiF signal is observed compared to the C-F group, suggesting the generation of LiF, which effectively improves the ionic conductivity of the SEI layer. Moreover, high-resolution I 3*d* spectra show two prominent peaks at 619.75 and 631.90 eV, corresponding to the I 3*d*_5/2_ and I 3*d*_3/2_ level of LiI, confirming the formation of LiI component in the SEI layer after injection treatment, highlighting the participation of I_3_^−^/I^−^ redox couple in regulating the electrode/electrolyte interface composition, which contributes to form a stable, robust, and LiF/LiI-rich SEI layer on the surface of graphite anode [50–52]. The reconstruction of SEI layer composition by the I_3_^−^/I^−^ redox couple is also can be further verified by the SEM and energy-dispersive X-ray spectroscopy (EDS) mapping images of S-Gra and R-Gra anodes. In detail, S-Gra displays high F atomic content (7.88%) due to the decomposition of LiPF_6_, and high O atomic content (7.25%) associated with the accumulation of Li_2_O (Figs. S9 and S10). After injection treatment, the O atomic content of R-Gra decreases to 5.62%, while the I atomic content increases to 0.2% (Figs. S11 and S12), manifesting the introduced iodine species are involved in the reaction for dissolving the Li_2_O by-products.

### Phase Structure and Composition Analysis of S-LFP and R-LFP

**Fig. 3:**
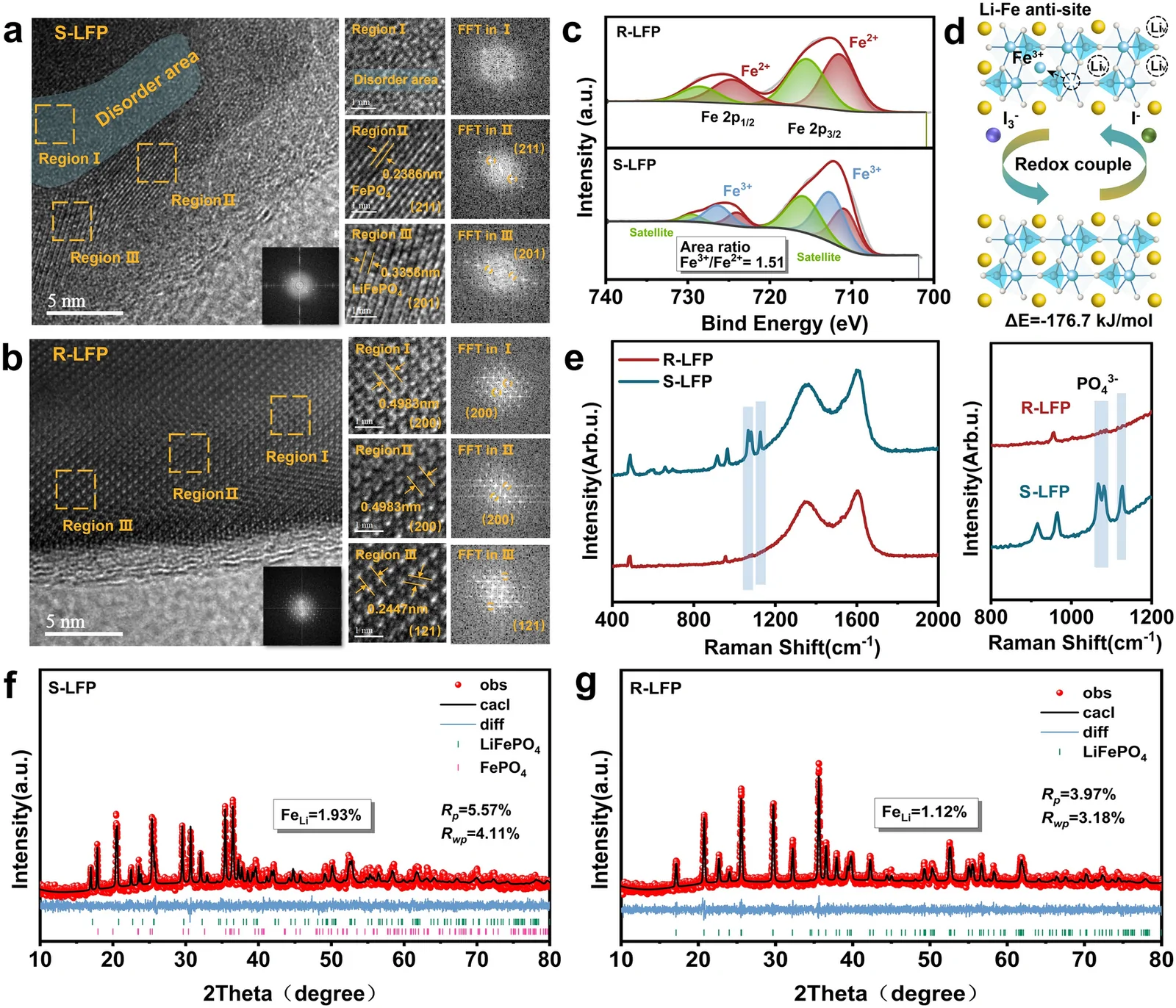
HRTEM images and the corresponding FFT electron diffraction patterns of **a** S-LFP and **b** R-LFP samples. **c** High-resolution Fe 2*p* spectra of S-LFP and R-LFP samples. **d** Schematic illustration of the regeneration. **e** Raman spectra of S-LFP and R-LFP samples with a magnified view highlighting key vibrational modes. Rietveld refinement results of **f** S-LFP and **g** R-LFP samples

To assess the feasibility of the I_3_^−^/I^−^ redox couple strategy, the phase structures and composition analysis of spent LFP (S-LFP) and regenerated LFP (R-LFP) cathodes were comprehensively investigated. Table S2 summarizes the elemental composition content and molar ratios of R-LFP and S-LFP samples based on ICP-OES analysis, demonstrating an apparent lithium loss in S-LFP, which not only contributes to the capacity fading, but also causes the partial Fe(III) emergence, and irreversibly impacts the electrode material performance [53]. The fast Fourier transform (FFT) electron diffraction pattern of S-LFP displays an irregular arrangement, indicating the attendance of structural degradation. Subsequently, high-resolution transmission electron microscope (HRTEM) was performed to detect the phase structure at the microscopic level. Three obvious regions (regions I-III highlighted in yellow boxes) were selected for further analysis, and each region displayed different lattice fringes and FFT patterns. The HRTEM image of Region I demonstrates the occurrence of disordered areas on the surface of S-LFP particle, suggesting the obstructed Li^+^ diffusion pathways hamper the transition from the FePO_4_ to LiFePO_4_, which results in the structural degradation after long-term cycling. Region II, located near the S-LFP particle edge, displays a lattice spacing of 0.2386 nm, corresponding to the (211) lattice plane of FePO_4_ (Fig. 3a). Meanwhile, the HRTEM image of Region III exhibits a lattice spacing of 0.3358 nm, corresponding to the (201) lattice plane of LiFePO_4_. The FFT patterns of Region III also reveals the diffraction spot of the FePO_4_ phase, confirming the coexistence of FePO_4_ and LiFePO_4_ phases. In contrast, the R-LFP cathode, analyzed from the surface to the interior, exhibits uniform phase distribution. The FFT patterns from the selected three regions display the close lattice spacings of 0.4983 and 0.2447 nm, corresponding to the (200) and (121) lattice planes of LiFePO_4_, respectively (Fig. 3b), suggesting a single-phase and homogeneous structure in R-LFP electrode. The high-resolution Fe 2*p* spectra were conducted to investigate the valence state of Fe in different LFP samples. For the S-LFP, the main peaks at 712.65 and 711.15 eV are ascribed to Fe(III) and Fe(II) in the Fe 2*p*_3/2_ region, corresponding to the phase of FePO_4_ and LiFePO_4_, respectively, suggesting the mixed-phase structure of S-LFP after long-term cycling (Fig. 3c). Quantified from the Fe 2*p*_3/2_ fitting peak area, the Fe(III)/Fe(II) area ratio is calculated as 1.51, further implying the dominant phase of FePO_4_ in S-LFP. For the R-LFP, the Fe^3+^ peak disappears from the high-resolution Fe 2*p* spectra, confirming that the FePO_4_ phase is fully converted into LiFePO_4_ after the regeneration treatment [54], which is schematically illustrated in Fig. 3d. Detailedly, the I_3_^−^/I^−^ couple is served as redox mediator to activate the dead lithium on the surface of graphite anode, facilitate the re-lithiation reaction of FePO_4_ in S-LFP, and reinforce the crystal structure integrity of LiFePO_4_ in R-LFP, thereby realizing the in situ regeneration of the LFP cathode in spent LFP/Gra pouch cells.

Moreover, the Raman spectra of S-LFP sample deliver three prominent peaks within 1000–1200 cm^−1^, which is attributed to the PO_4_^3−^ species (Fig. 3e), *i.e.*, FePO_4_ phase. After regeneration process, the characteristic peaks vanish in R-LFP sample and are consistent with these of bare LFP (B-LFP) sample (Fig. S13). That is to say, the LiFePO_4_ component is successfully replenished through the I_3_^−^/I^−^ redox couple strategy. In addition to the phase recovery, Li–Fe antisite defects also represent a critical parameter for the repair quality of S-LFP. As a result, Rietveld refinement XRD results of S-LFP sample exhibit an extremely ordered (Pnma) space group with both FePO_4_ and LiFePO_4_ phases (Fig. 3f), which is attributed to the presence of lithium vacancies, leading to the oxidation of Fe^2+^ to Fe^3+^ during cycling [23]. In contrast, the Rietveld refinement XRD results of R-LFP sample solely consist the LiFePO_4_ phase without detectable impurity phases (Fig. 3g), demonstrating lithium vacancies are rectified through the re-intercalation reaction of Li^+^ ions. Based on the refined data of lattice parameters in Tables S3 and S4, it can be observed the calculated defect concentration in R-LFP is 1.12%, which is higher than that in S-LFP (1.93%), confirming the reduction of Li–Fe antisite defects and the restoration of structural integrity in R-LFP after the regeneration treatment, which is vital for accelerating the diffusion of Li^+^ ions as well as the long-term cyclic stability of LFP pouch cells [55]. Additionally, after carefully comparing the SEM and corresponding EDS mapping images of R-LFP with S-LFP sample (Figs. S14–S16), there are no obvious cracks on the surface of R-LFP cathode, and it remains smooth and dense without any noticeable deposits, testifying the nondestructive restoration feature of I_3_^−^/I^−^ redox couple strategy without chemical side reactions between the injected iodine species and electrode materials [56]. Furthermore, DFT calculations were conducted to evaluate the reaction energies between I^−^ ions and FePO_4_ (3I^−^ + 2FePO_4_ + 2Li^+^ = I_3_^−^ + 2LiFePO_4_, ΔE = − 375.3 kJ mol^−1^). The exothermic nature suggests the reaction spontaneity of the regeneration of FePO_4_ component in S-LFP cathode by I_3_^−^/I^−^ redox couple. In brief, this direct LFP regeneration technique based on the I_3_^−^/I^−^ redox couple, effectively facilitates the full use of dead lithium, the spontaneous structural integrity reconfiguration, and the sufficient elimination of Li–Fe antisite defects in R-LFP, demonstrating its multiple feasibility for repairing the S-LFP cathode.

### Intrinsic Action Mechanisms of the I_3_^−^/I^−^ Redox Couple Strategy

**Fig. 4:**
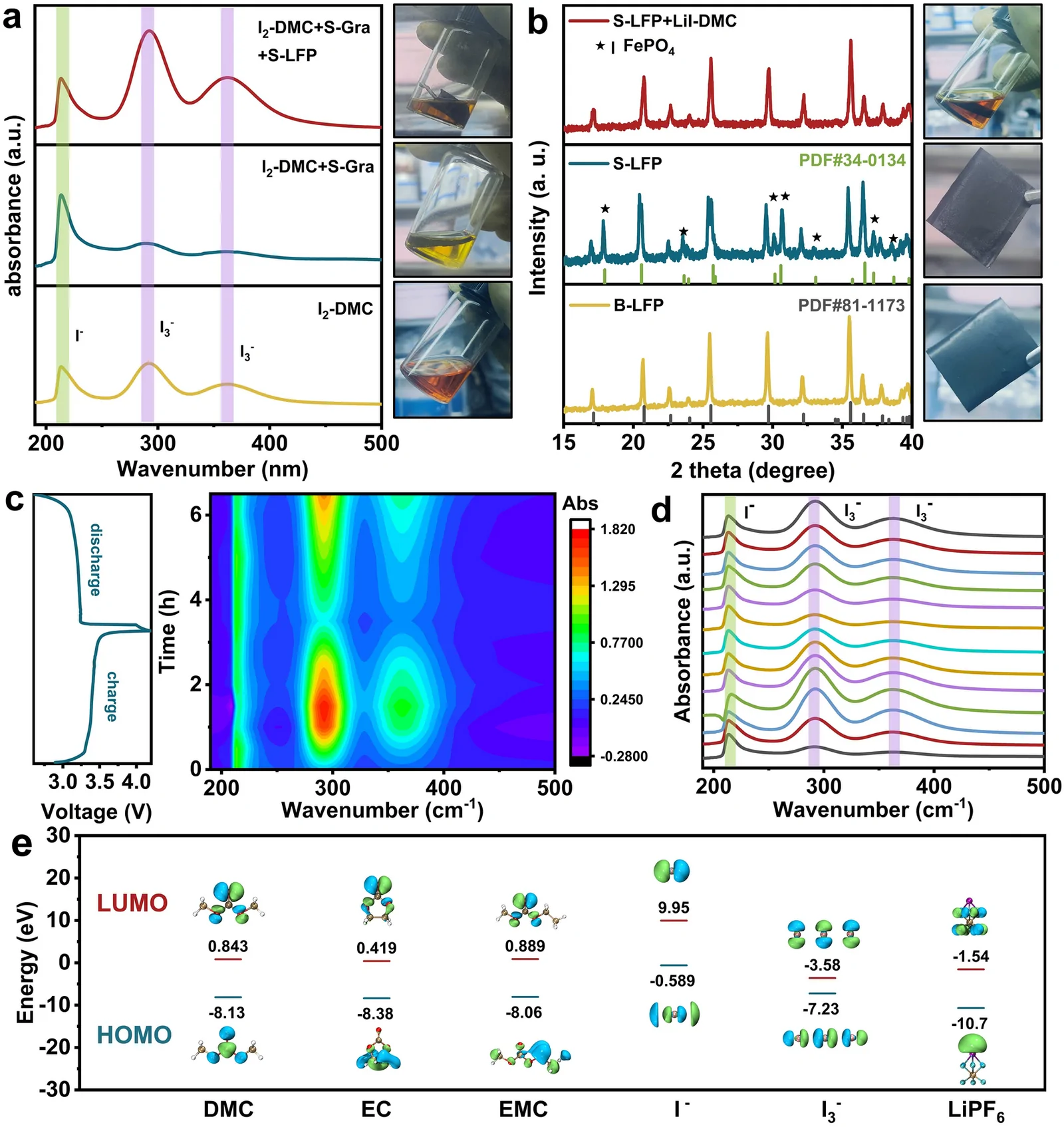
**a** UV–Vis spectra and the corresponding color changes of I_2_-DMC solution containing the S-LFP and S-Gra electrodes.** b** XRD patterns and digital photos of B-LFP, S-LFP and S-LFP cathodes soaked in LiI-DMC solution; **c, d** In-situ UV–Vis spectra of the S-LFP/S-Gra cells during the regeneration process by the recovery reagent. **e** LUMO–HOMO energy levels of different solutes and solvents

Ultraviolet spectroscopy (UV–Vis) was conducted to further validate the action mechanism of the I_3_^−^/I^−^ redox couple strategy owing to the high detection sensitivity of ultraviolet light for iodine species in the solution. As presented in Figs. 4a and S17, the characteristic peak of I^−^ (215 nm) and the characteristic peaks of I_3_^−^ (288 and 360 nm) can be clearly observed in the I_2_-containing DMC (I_2_-DMC) solution [36, 38], which is the coexistence form of elemental iodine in polar solvent, while only the I^−^ peak is detected in LiI-containing DMC (LiI-DMC) solution [42]. After introducing S-Gra into the I_2_-DMC solution, the intensity of I^−^ peak strengthen, the intensity of I_3_^−^ peaks significantly weaken, and the initial reddish-brown I_2_-DMC solution changes to bright yellow, which can be ascribed to the spontaneous reaction within dead lithium/Li_2_O and I_3_^−^ species. when the S-LFP cathode is also soaked in the S-Gra-reacted I_2_-DMC solution, the intensity of I^−^ peak decreases, while the intensity of I_3_^−^ peaks recover owing to the oxidization of I^−^ to I_3_^−^ species, induced by the spontaneous reaction between I^−^ ions and FePO_4_. Likewise, the color of the corresponding solution reverts to the characteristic reddish-brown of initial I_2_-DMC solution. To further verify the lithiation process of S-LFP by the I_3_^−^/I^−^ redox couple, the XRD patterns of B-LFP, S-LFP and S-LFP cathodes soaked in LiI-DMC solution were conducted. As shown in Fig. 4b, S-LFP exhibits the coexistence of FePO_4_ and LiFePO_4_ phase. After fully soaking in LiI-DMC solution, there is no diffraction peaks belonging to FePO_4_, suggesting a phase conversion from FePO_4_ to LiFePO_4_ driven by the LiI component. These findings collectively demonstrate the efficient and reversible operation of this redox couple, enabling the regeneration of Li^+^ ions and restoration of spent electrode materials through spontaneous lithiation and redox process, which can be convincingly verified by the in situ UV–Vis spectra. As displayed in Fig. 4c, d, an obvious intensity rise can be detected in I_3_^−^ peak during the charge process when the voltage exceeds 2.9 V, corresponding to the oxidation of I^−^ to I_3_^−^. As the continuous increase of charge voltage, the I_3_^−^ peak gradually weakens while the I^−^ peak appears at the end of charge process, which is ascribed to the ongoing removal of dead lithium, the regeneration of I^−^, and the concurrent modification of SEI layer by I_3_^−^. During the discharge process, a notable elevation in I_3_^−^ peak is observed, confirming I^−^ ions spread to the cathode side, and subsequently oxidized to I_3_^−^ species. This highly reversible redox process enables the lithiation of S-LFP cathode and the reactivation of dead lithium on S-Gra anode. Furthermore, the frontier molecular orbital theory was employed to evaluate the energy levels of the lowest unoccupied molecular orbital (LUMO) and the highest occupied molecular orbital (HOMO) for I^−^ and I_3_^−^ (Fig. 4e). Among the electrolyte components, I^−^ exhibits the highest LUMO energy level (9.95 eV) compared to DMC/EC/EMC molecules (0.419–0.889 eV) and PF_6_^−^ anions (− 1.54 eV), which is thermodynamically favorable for the oxidation of I^−^ to I_3_^−^ on the cathode side, minimizing the oxidative degradation of solvent molecules. Similarly, I_3_^−^ holds beneficial effect on the anode side owing to the weaker HOMO energy level (− 7.23 eV) than solvent molecules (+ 8.06 ~ − 8.38 eV), effectively contributing to reactivate dead lithium into the delithiated cathode rather than the decomposition of solvents. These results validate the dual role of I_3_^−^/I^−^ redox couple in stabilizing the electrolyte environment reactivating the dead lithium to support the cyclic sustainability.

To verify the impact of iodine species on electrode corrosion, CV curves of Li||Al cells were performed in raw LiPF_6_ electrolyte and I_2_-containing LiPF_6_ electrolyte. The highest oxidation current appears in the initial cycle and gradually decreases in the subsequent cycles (Fig. S18), implying the formation of passivation layer on the Al surface, which can be mainly attributed to the electrolyte decomposition. The pits in SEM images confirms the corrosion of Al foil after CV test (Fig. S19), and there is almost no difference between raw and I_2_-containing LiPF_6_ electrolytes, verifying the introduction of iodine does not induce extra electrode corrosion. Similar evidences can be also observed in the XPS spectra and XRD patterns of Al foil after CV test. According to the high-resolution F 1*s* and Al 2*p* spectra, an apparent AlF_3_ passivation layer on Al surface can be ascribed to the decomposition and corrosion of LiPF_6_ component (Fig. S20). Meanwhile, the XRD patterns of Al foils after CV test shows consistent characteristic peaks in both raw and I_2_-containing LiPF_6_ electrolytes (Fig. S21), comprehensively demonstrating extremely weak corrosion effect of iodine species on Al electrode. In addition, SEM images of Cu foil after soaking in the raw and I_2_-containing LiPF_6_ electrolytes also deliver extremely minor differences in microscopic morphology (Fig. S22), suggesting no potential corrosion of I_2_/I_3_^−^ species on Cu current collector. Based on the above results, iodine injection reagent effectively removes dead lithium and optimizes the SEI layer on the surface of S-Gra, while S-LFP receives Li^+^ to complete its regeneration process without the electrochemical corrosion on current collectors, electrode binders, and cell casing materials. This innovative approach operates without the external additives, enabling a closed-loop, non-disassembly regeneration strategy for spent, fully assembled cells. These findings underscore the potential for sustainable and efficient battery recycling, promoting material recovery and reuse within a fully integrated process.

### Electrochemical and Practical Evaluation of Pouch Cells Before and After Injection

**Fig. 5:**
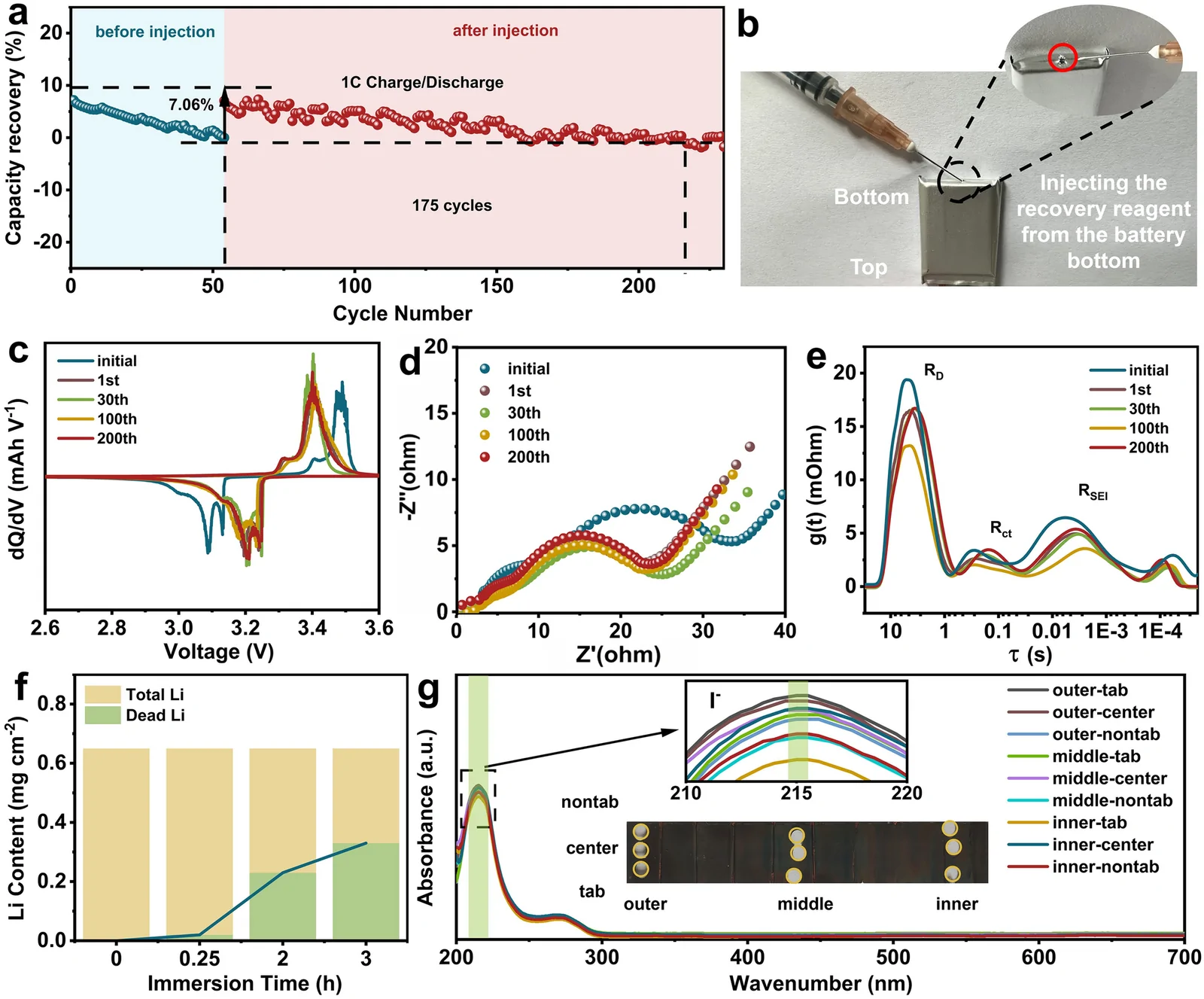
**a** Capacity recovery ratio of pouch cells before and after injecting the I_2_-containing recovery reagent at 1C. **b** Schematic diagram for injection of recovery reagent from bottom of the spent pouch cells. **c** dQ/dV plots, **d** EIS spectra and **e** the corresponding DRT spectra of pouch cells before and after injection. **f** Dead Li content test of S-Gra anode in I2-DMC solution. **g** UV–vis spectra collected from various locations of R-Gra anode

The I_2_-containing recovery reagent was injected into the spent pouch cells to evaluate the feasibility and effectiveness of I_3_^−^/I^−^ redox couple strategy in recovering the reversible capacity. As displayed in Fig. 5a, b, the injected cell exhibits a capacity recovery ratio of 7.06%, and decrease to the capacity level of the untreated cell after 175 cycles, which suffers from fast capacity decay within only 54 cycles, highlighting the notable capacity recovery and preferable cyclic stability of the injection strategy. To ensure battery safety, the pinhole in the post-injection pouch cells was tightly sealed by termination tape (lithium battery termination tape, Shenzhen Kejing Zhida Technology Co., Ltd.) with elastomeric adhesive characteristic, which is chemically inert toward electrolyte components. Owing to the strong mechanical adhesion and stable chemical compatibility, the sealing tape layer can remain structurally intact, and no potential leakage pathway or liquid electrolyte seepage are detected throughout the subsequent electrochemical testing process. As demonstrated, Fig. S23 contrasts the appearance of tape sealed post-injection LFP pouch cell after 500 cycles with the normal LFP pouch cell, and the insignificant differences indicate the termination tape sealing strategy can effectively address the battery safety issue. Notably, Fig. S24 presents the capacity recovery ratio and EIS spectra of spent pouch cells before and after injecting commercial LiPF_6_ electrolyte without iodine species. The continuously decreasing capacity recovery rate and the almost overlapping electrochemical impedances demonstrate the critical role of I_3_^−^/I^−^ redox couple in I_2_-containing recovery reagent, which can effectively activate the dead lithium on the surface of S-Gra anode and SEI layer.

The differential capacity (dQ/dV) curves reveal a significantly reduced overvoltage of ~ 0.2 V before and after injection treatment (Fig. 5c), which can maintain for 200 cycles, indicating fast and stable interface reaction kinetics activated by the I_3_^−^/I^−^ redox couple. Correspondingly, the internal resistances of the injected pouch cells are much lower than these of the spent pouch cells (Fig. 5d), implying the fast charge transfer behavior driven by I_3_^−^/I^−^ redox couple. Meanwhile, the two semicircles in EIS spectra corresponding to the charge transfer resistance (*R*_*ct*_) and SEI resistance (*R*_*SEI*_) are nearly coincident after 100 cycles, verifying the formation of an uniform and dense SEI layer. To elucidate the resistance changes, the distribution of relaxation time (DRT) curves was derived from the EIS spectra. Three peaks within 10–1 s, 1–10^−2^ s, and 10^–2^ to 10^–3^ s (Fig. 5e), respectively, correspond to the diffusion resistance (*R*_*D*_), the charge transfer resistance (*R*_*ct*_), and the SEI resistance (*R*_*SEI*_) [57]. After injection treatment, these three resistances gradually decrease with the increase of cycle number, while *R*_*D*_ exhibits the most obvious decline (Fig. S25), which can be attributed to the full activation of dead lithium on the surface of graphite anode, which always blocks the ionic diffusion path and hinders the ionic transport behavior [58]. To address concerns regarding potential self-discharge associated with the I_3_^−^/I^−^ redox couple [42], the open-circuit voltage (OCV) decay rate was measured in pouch cells before and after injection. Following a full charge at 1C and one week of rest, the injected cells exhibited a significantly lower OCV decay rate (0.001) compared to the uninjected cells (0.0027, Fig. S26a), indicating that self-discharge was effectively suppressed owing to the formation of robust protective SEI layer. Moreover, the enhanced interfacial kinetics after injection treatment led to improved rate performance. As illustrated in Fig. S26b, the injected cells maintained high-capacity recovery across various charge/discharge rates, further demonstrating the effectiveness of the I_3_^−^/I^−^ redox couple in performance enhancement. To investigate the effect of I_2_ species as an electrolyte additive, the cycling performances of LFP pouch cells were tested in both raw and I_2_-containing LiPF_6_ electrolytes. As delivered in Fig. S27, the I_2_-containing LiPF_6_ electrolyte enabled significantly higher capacity retention (94.7% after 200 cycles), in contrast to the 84.6% retention observed with raw LiPF_6_ electrolyte, confirming the positive contribution of iodine redox couple on the cyclic stability. Meanwhile, EIS spectra of LFP pouch cells in different electrolytes further revealed the consistently lower interfacial resistances (*R*_*D*_/*R*_ct_/*R*_SEI_) in I_2_-containing LiPF_6_ electrolyte, demonstrating the key role of I_2_ additive in promoting the electrochemical performances (Fig. S28). Therefore, the I_3_^−^/I^−^ redox couple can not only serve as regeneration agent to reactivate the loss capacity of degraded LFP cells in this work, but also act as electrolyte additive to stabilize the battery performance. The dead lithium content in S-Gra anode was analyzed by ICP-OES through immersing it in I_2_-DMC solution. As presented in Fig. 5f, the total lithium content in S-Gra anode gradually decreases with the increasing immersion time, while the dead lithium content delivers a linear relationship with the square root of immersion time (Fig. S29a), indicating a one-dimensional diffusion mechanism, in which lithium replenishment originates from the electrode surface. The complementary UV–Vis spectroscopy results reveal a sharp decrease in the intensity of I_3_^−^ characteristic peak after the immersion of S-Gra anode (Fig. S29b), confirming the reaction of dead lithium in S-Gra with the I_3_^−^ species in I_2_-DMC solution. To further explore the penetration behavior of the recovery reagent into the interior of pouch cells, UV–Vis spectra were collected from different positions of the R-Gra anode. As summarized in Fig. 5g, characteristic absorption peak of I^−^ species was detected, which may be originated from the reaction between dead lithium in S-Gra and I_3_^−^ component in recover reagent (2Li + I_3_^−^ → 2Li^+^  + 3I^−^). Meanwhile, the UV–Vis spectra collected from various positions of R-Gra anode hold the characteristic peak of I⁻, demonstrating the iodine-based recovery reagent fully penetrates the pouch cells and activates the dead lithium component throughout the S-Gra anode, which is beneficial for the full activation and direct regeneration of spent LFP/Gra pouch cells.

### Electrochemical Evaluation of Regeneration Effectiveness in Pouch Cells with Different Degradation Degrees

**Fig. 6:**
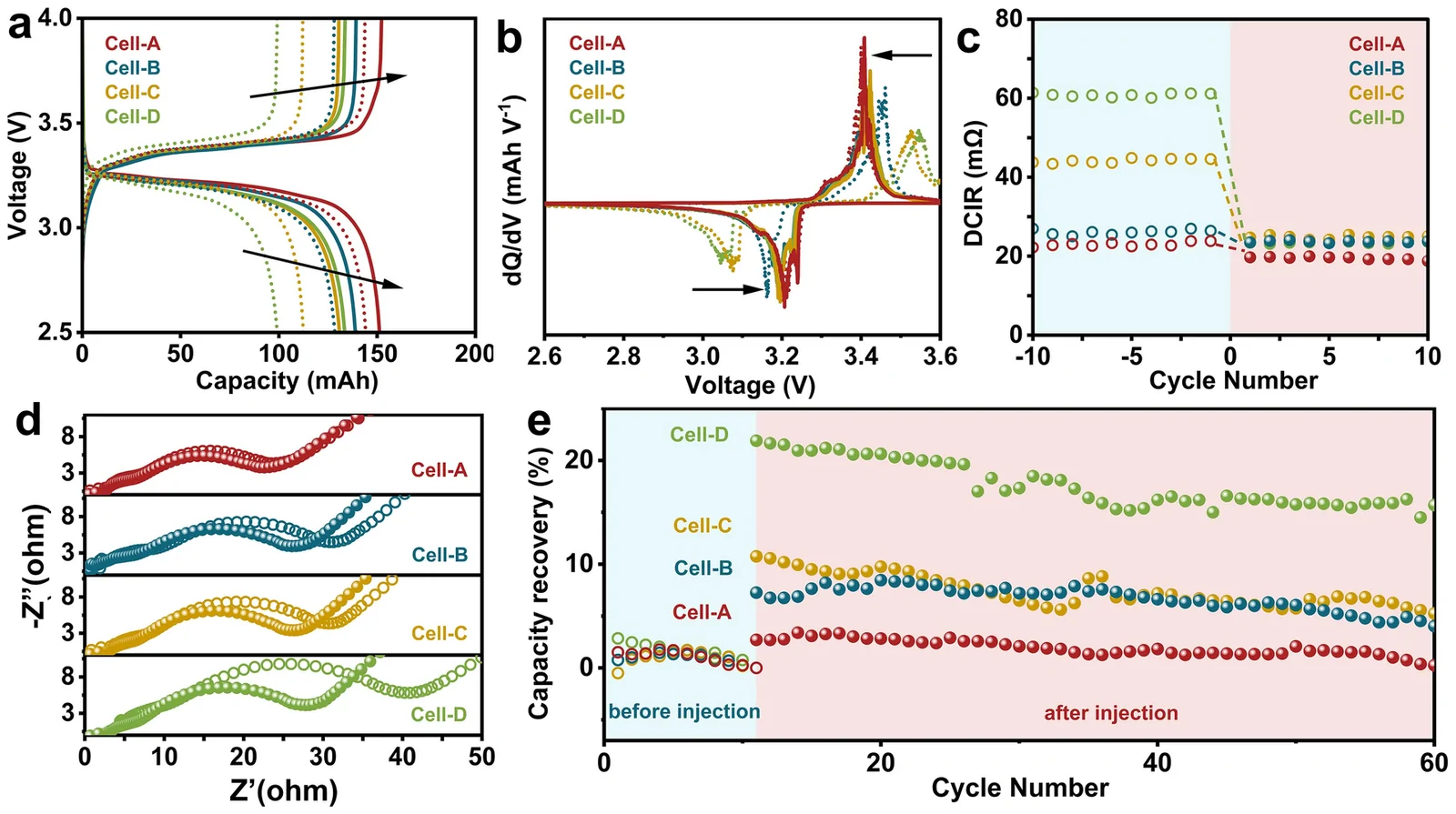
**a** Constant current charge/discharge curves of pouch cells with different degradation degrees at 1C before (dashed lines) and after (solid lines) injection treatment. **b** Differential capacity (dQ/dV) plots of pouch cells with different degradation degrees before (dashed lines) and after (solid lines) injection treatment. **c** DC internal resistances (DCIR) of pouch cells with different degradation degrees before (hollow balls) and after (solid balls) injection treatment; **d** EIS spectra of pouch cells with different degradation degrees before (hollow balls) and after (solid balls) injection treatment. **e** Capacity recovery ratio of pouch cells with different degradation degrees at 1C before (hollow balls) and after (solid balls) injection treatment

To verify the feasibility of I_2_-containing recovery reagent for different battery states of health (SOH), four pouch cells with different degradation degrees, respectively, marked as Cell-A, Cell-B, Cell-C, and Cell-D, were selected as study object to reveal their electrochemical performance differences before and after injection treatment (Table S5). As delivered in Fig. 6a, the remaining discharge capacities of Cell-A, Cell-B, Cell-C, and Cell-D are 145.8, 128.4, 113.6, and 99.8 mAh, respectively, manifesting the gradually increasing degradation degree. After injection treatment, the corresponding discharge capacity can be recovered to 151.19, 139.86, 130.8, and 133.99 mAh, respectively. Similarly, according to the differential capacity (dQ/dV) plots of these pouch cells, it can be observed the corresponding overvoltage, *i.e.*, the gaps between the oxidation potential and the reduction potential, is greatly reduced after injection treatment (Fig. 6b). As typical sample, the overvoltage of Cell-D before and after injection treatment is 0.5 and 0.22 V, respectively, and the narrowing voltage gap is beneficial for accelerating the electrode reaction kinetics and enhancing the energy efficiency of the battery. Figure 6c presents the DC internal resistances (DCIR) of these pouch cells, all the DCIR values decrease accompanying with the injection of I_2_-containing recovery reagent, indicating the recovery of battery reaction activity.

Figure 6d further contrasts the differences of these pouch cells in AC impedances, and the noticeable resistance reductions in the regenerated pouch cells, especially in Cell-B/Cell-C/Cell-D, can be attributed to the activation of deal lithium and the reconstruction of SEI layer, contributing to fast interface charge transfer behavior. Owing to the merits of small voltage polarization and charge transfer resistances after injecting I_2_-containing recovery reagent, the regenerated pouch cells of Cell-A, Cell-B, Cell-C, and Cell-D, display initial capacity recovery ratios of 3.38%, 7.25%, 10.75%, and 21.64%, respectively (Fig. 6e). An interesting phenomenon is that the injection of recovery reagent has the most significant improvement effect on the performance of Cell-D (with the highest degradation level), implying the effectiveness of this injection strategy is relatively dependent on the content of inactive lithium in the spent pouch cells. As verified by the ICP-OES results in Table S6, Cell-D also holds the highest content of inactive lithium, which mainly exists on the surface of spent graphite anode as the form of dead metallic lithium or inert lithium compounds (Li_2_O). These results highlight the spent pouch cells with more pronounced deterioration degree and higher dead lithium content, exhibit more Li^+^ ions activated by iodine species, as well as more obvious capacity recovery after injection treatment, and the degradation status of spent punch cells is also of crucial importance in the selection of battery recycling strategy. The successful application of this injection strategy further demonstrates its significant advantages and application potential for large-scale regeneration of spent pouch cells with different degraded levels.

### Economic and Environmental Analysis of Different Battery Recycling Technologies

**Fig. 7:**
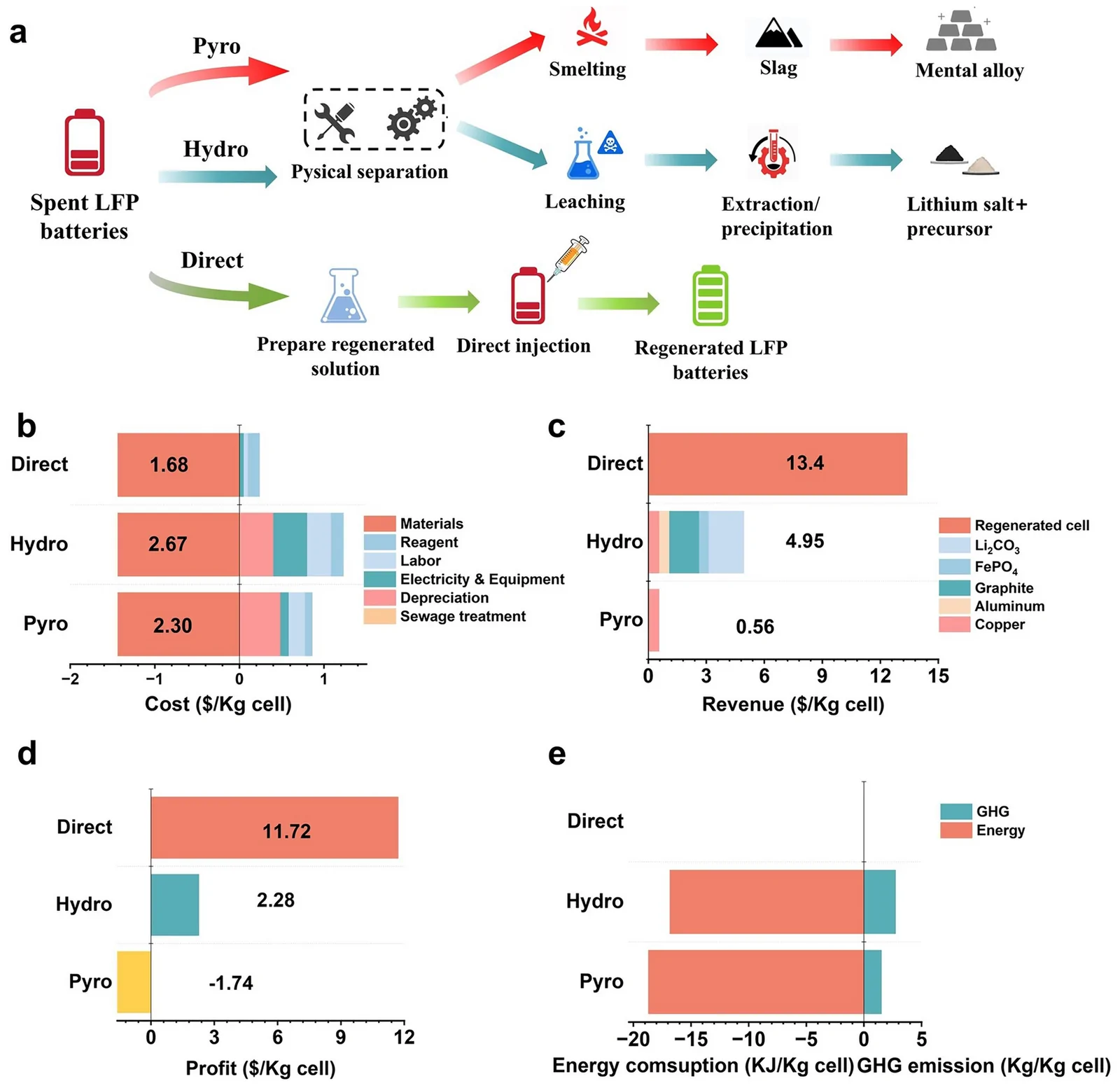
**a** Schematic illustration of three recycling routes: pyrometallurgical (pyro), hydrometallurgical (hydro), and direct regeneration.** b** Cost comparison, **c** revenue analysis, and **d** profit evaluation for each recycling route applied to degraded LFP batteries. **e** Energy consumption and greenhouse gas (GHG) emissions associated with the different recycling processes

Combining material flow analysis from the Everbatt 2023 framework, we conducted a comprehensive evaluation of the economic and environmental impacts of recycling 1 kg of degraded LFP batteries. Figure 7a illustrates the three major recycling technologies currently, *i.e.*, pyrometallurgical (pyro), hydrometallurgical (hydro), and direct regeneration. Among them, the direct route offers a notably simpler process compared to the pyro and hydro methods. Figure 7b–d presents a comparison of cost, revenue, and profit for the three strategies. Due to the complexity of powder recovery and resynthesis, the cost per kilogram of battery for the pyro and hydro processes reaches $2.30–2.67 (excluding government subsidies), whereas the cost for the direct strategy is significantly lower at $1.68, with reagent (electrolyte) costs comprising a substantial portion of the total (Table S7). Revenue generation is a key metric in evaluating recycling technologies. As shown in Fig. 7c, the pyro process yields only about $1.25 kg^−1^, primarily from recovered copper, with other valuable components discarded as slag (Table S8). The hydro process offers slightly better revenue, mostly from Li_2_CO_3_ and copper recovery. In contrast, the direct strategy results in the regeneration of complete battery cells, eliminating disassembly and significantly enhancing revenue potential. Without government subsidies, the pyro and hydro processes yield negative and marginal profits, respectively (Fig. 7d). The direct regeneration strategy, however, achieves a profit of $11.72 kg^−1^ of regenerated batteries, highlighting its superior economic viability (Table S9). Environmental metrics further underscore the advantages of the direct approach. The pyro and hydro processes consume 18.72 and 16.85 MJ kg^−1^ of energy, respectively, while the direct strategy, driven by iodine redox reactions, requires no external energy input (Fig. 7e). Likewise, greenhouse gas (GHG) emissions for pyro and hydro routes are 1.53 and 2.76 kg CO_2_-eq per kg of battery, respectively. In contrast, the direct process results in no GHG emissions during operation (Table S10), reinforcing its environmental benefits. Overall, compared to conventional methods, the direct regeneration strategy stands out as a highly competitive and sustainable solution for LFP battery recycling—economically advantageous due to the high value of regenerated cells, and environmentally superior due to its energy-free and zero-emission process [59].

## Conclusions

In summary, we showcase a novel strategy for regenerating the capacity of S-LFP batteries through the direct injection of a recovery reagent based on the I_3_^−^/I^−^ redox couple. By employing multiple characterization techniques, we comprehensively analyzed the electrochemical behavior occurring on both the cathode and anode during the operation of the I_3_^−^/I^−^ redox couple and elucidated the underlying mechanisms. Our findings highlight that the core of the regeneration process lies in the shuttle effect of the I_3_^−^/I^−^ redox couple, which delivers three key benefits: (1) optimization of the SEI and reactivation of dead lithium: The I_3_^−^ species reactivates dead lithium on the graphite anode, serving as a lithium source for replenishment; (2) elimination of Li–Fe antisite defects: The generated I^−^ ions act as carriers of Li^+^, spontaneously reacting with delithiated LFP to effectively remove Li–Fe antisite defects; and (3) enhanced penetration and uniform recovery: The unique properties of pouch cells facilitate thorough penetration of the recovery reagent, ensuring comprehensive regeneration within the cell. The pouch cells regenerated through this method display remarkable capacity recovery, significantly improved kinetics, and enhanced cyclic stability, effectively prolonging their service life. This proposed strategy not only streamlines the recycling process by avoiding cell disassembly but also preserves the structural integrity of the cells. By offering an efficient and cost-effective pathway to extend the service life of S-LFP batteries, this approach provides a promising solution for sustainable battery management and contributes to advancing circular energy systems.

## Supplementary Information

Below is the link to the electronic supplementary material.

Supplementary file1 (DOCX 6686 kb)
