# Supplementary material for: Bioinspired Injection Therapy for Spent LiFePO4 Batteries: A Non-Invasive Strategy for Capacity Regeneration and Longevity Enhancement
Source: Nanomicro Lett. 2026 Feb 9;18:245. doi: 10.1007/s40820-026-02091-1 (PMC12886631; doi:10.1007/s40820-026-02091-1)
Supplement: Supplementary file 1 — Supplementary file1 (DOCX 6686 kb) [file 40820_2026_2091_MOESM1_ESM.docx]

Supporting Information for

**Bioinspired Injection Therapy for Spent LiFePO_4_ Batteries: A Non-Invasive Strategy for Capacity Regeneration and Longevity Enhancement**

Peng Wang ^1^, Jian Wang ^1^, Longwei Bai ^1^, Na Li ^1,^ *, Chuangcong Zhou ^2, 5^, Mingyang Chen ^2^, Jialiang Zhang ^3,^ *, Zhenyue Xing ^2^, Zaowen Zhao ^2^, Wei Zhang ^4,^ *, Xiaodong Shi ^2,^ *

^1^ Hebei Key Laboratory of Flexible Functional Materials, School of Materials Science and Engineering, Hebei University of Science and Technology, Shijiazhuang 050018, P. R. China

^2^ State Key Laboratory of Tropic Ocean Engineering Materials and Materials Evaluation, School of Materials Science and Engineering, Hainan University, Haikou 570228, P. R. China

^3^ State Key Laboratory of Advanced Metallurgy, University of Science and Technology Beijing, No. 30 Xueyuan Road, Haidian District, Beijing 100083, P. R. China

^4^ Christopher Ingold Laboratory, Department of Chemistry, University College London, London WC1H 0AJ, UK

^5^ College of Physics and Electronic Engineering, Nanyang Normal University, Nanyang 473061, P. R. China

* Corresponding authors. E-mail: [nali90@hebust.edu.cn](mailto:nali90@hebust.edu.cn) (Na Li); [jialiangzhang@ustb.edu.cn](mailto:jialiangzhang@ustb.edu.cn) (Jialiang Zhang); [wei.zhang.21@ucl.ac.uk](mailto:wei.zhang.21@ucl.ac.uk) (Wei Zhang); [shixiaodong@hainanu.edu.cn](mailto:shixiaodong@hainanu.edu.cn) (Xiaodong Shi)

**Supplementary Figures and Tables**


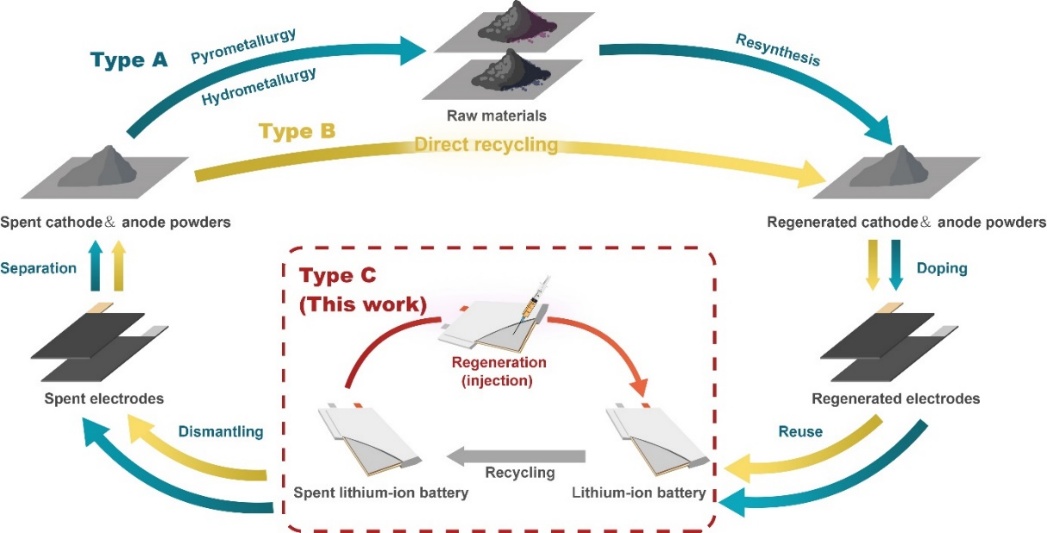


**Fig. S1** Schematic comparison of LFP battery recycling processes, highlighting conventional methods and the proposed strategy, where the direct recycling (Type C) eliminates the necessity to dismantling and separating processes and significantly reduces resource and energy use consumption and environmental impact.


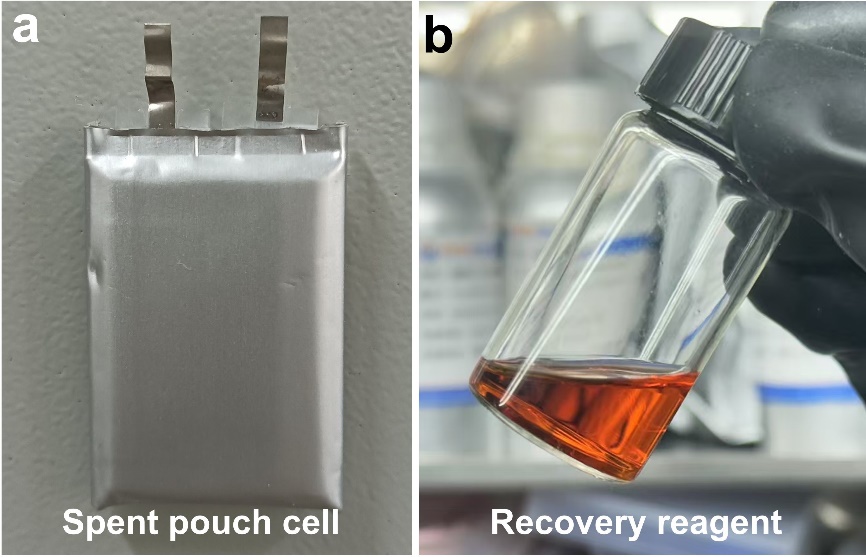


**Fig. S2** Digital photo of (**a**) spent LiFePO_4_/graphite (LFP/Gra) pouch cells and (**b**) the as-prepared I_2_-containing recovery reagent


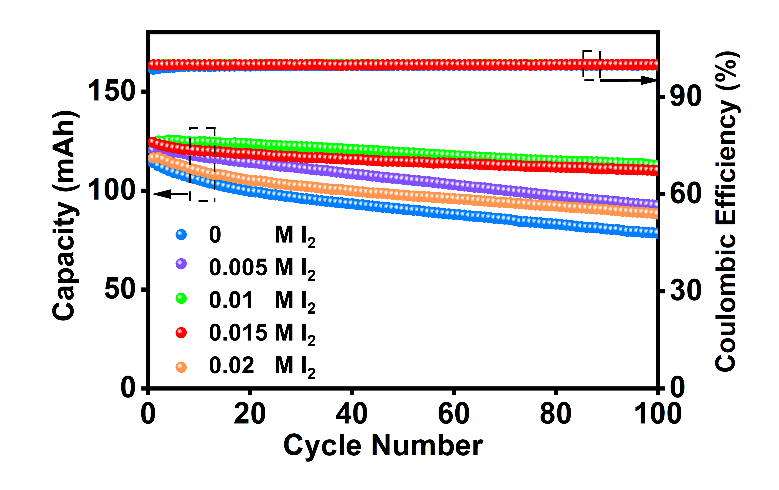


**Fig. S3** Cycling performances of spent LFP/Gra pouch cells in I_2_-containing electrolyte with different I_2_ molar concentrations (0-0.02 M)


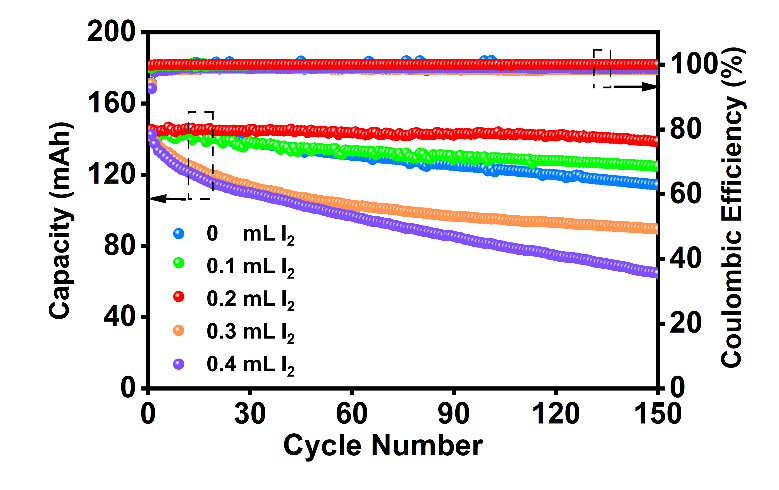


**Fig. S4** Cycling performances of spent LFP/Gra pouch cells in I_2_-containing electrolyte with different injection volumes


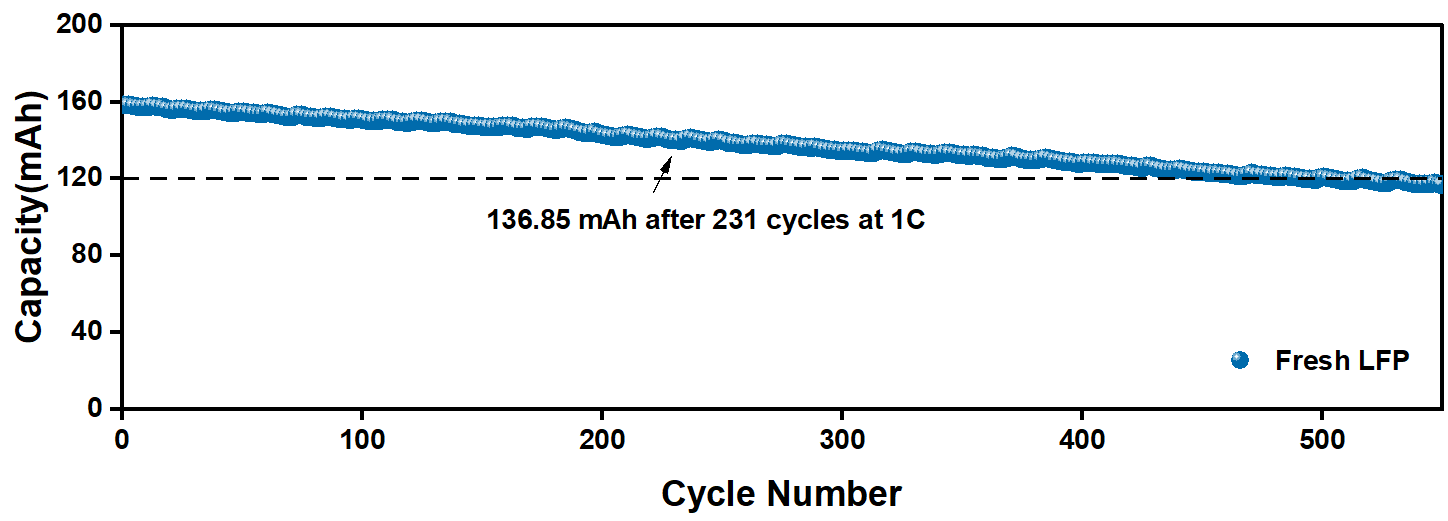


**Fig. S5** Cycling performances of fresh LFP pouch cells at 1C without the injection treatment of I_2_-containing recovery reagent


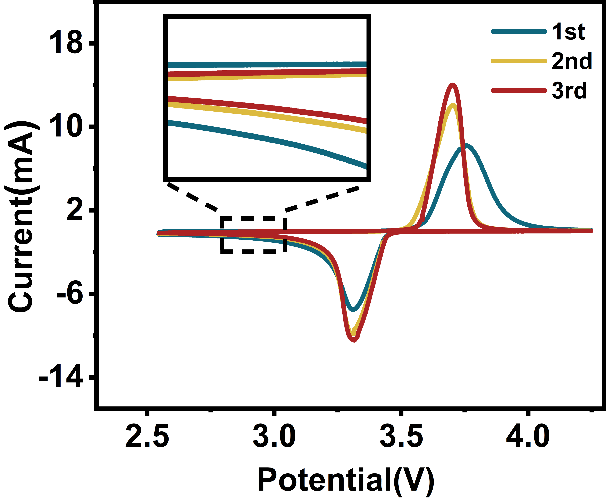


**Fig. S6** CV curves of spent LFP/Gra pouch cells in iodine-free electrolyte within 2.5-4.2 V at 0.1 mV s^-1^


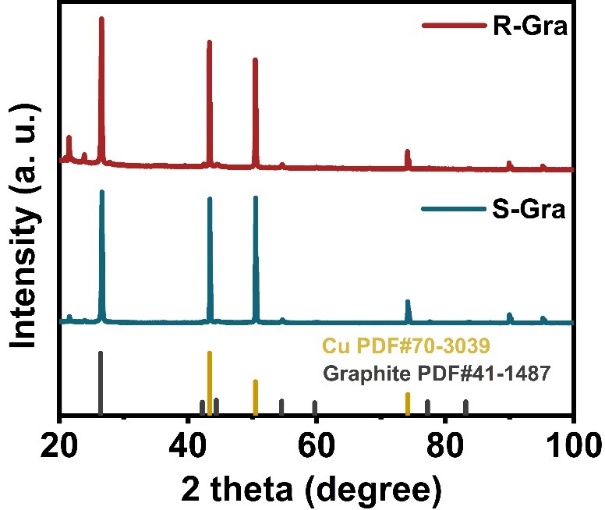


**Fig. S7** XRD patterns of S-Gra and R-Gra anodes


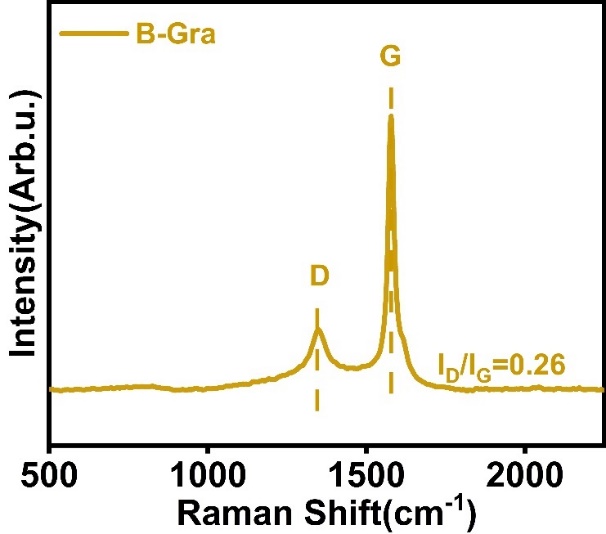


**Fig. S8** Raman spectra of B-Gra anode


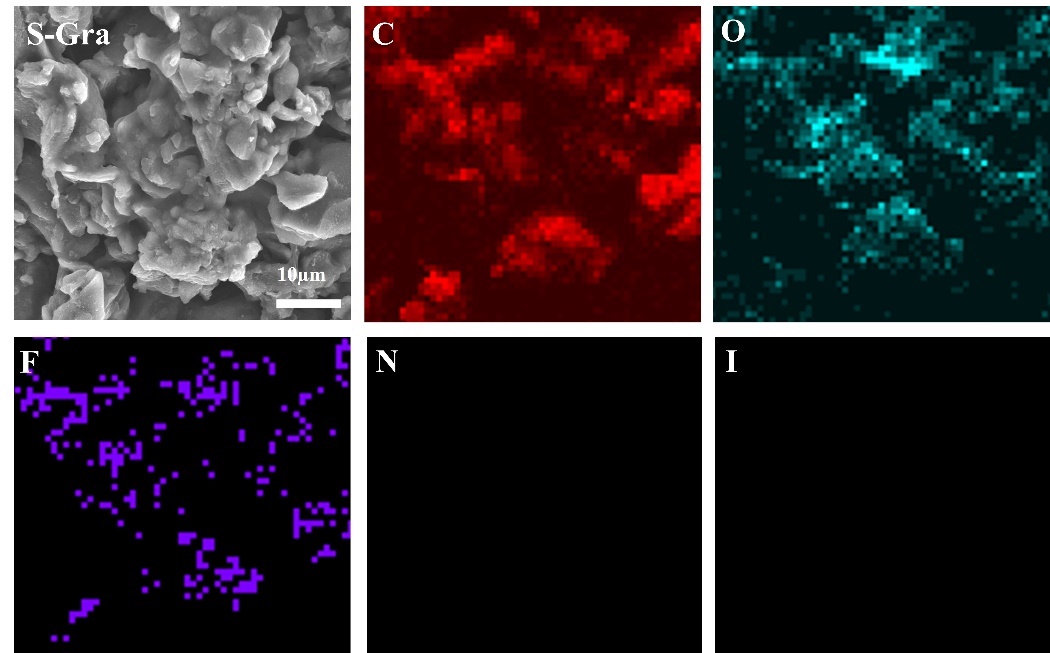


**Fig. S9** SEM images and EDS mapping images of S-Gra anode

**
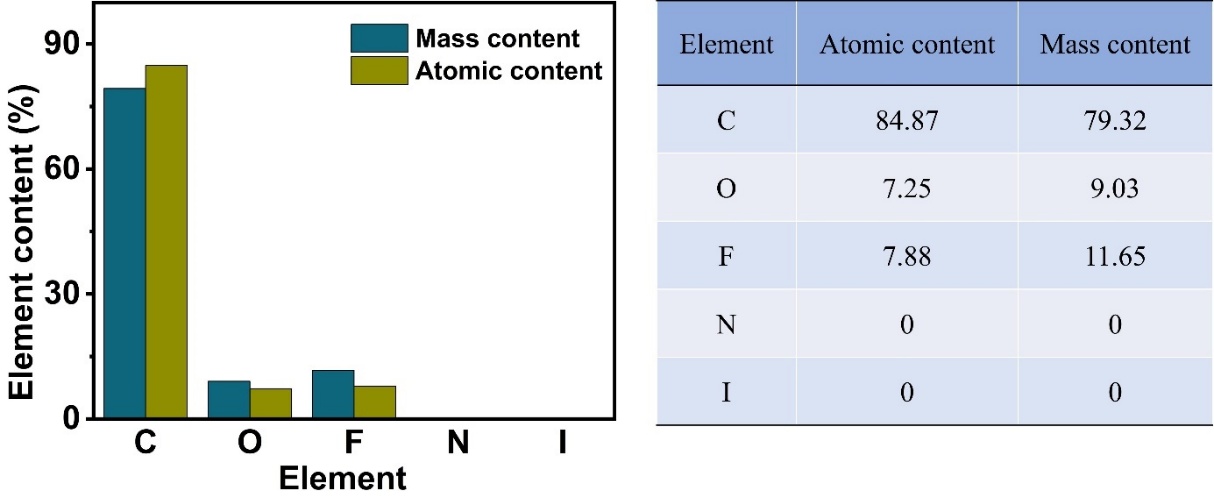
**

**Fig. S10** Atomic and mass content of C, O, F, N and I in S-Gra anode

**
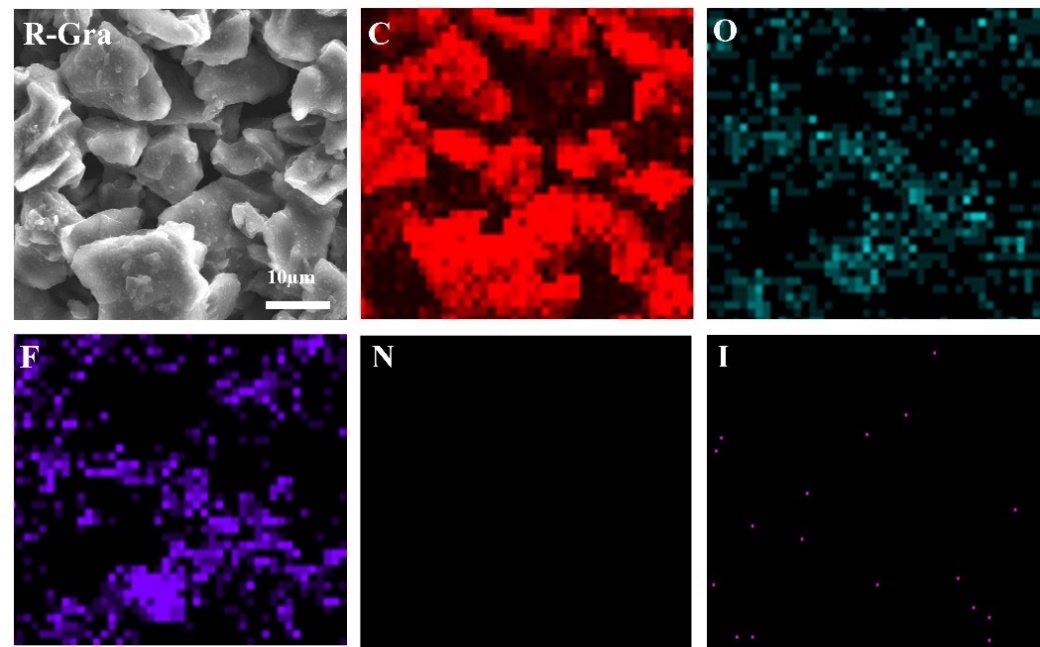
**

**Fig. S11** SEM images and EDS mapping images of R-Gra anode

**
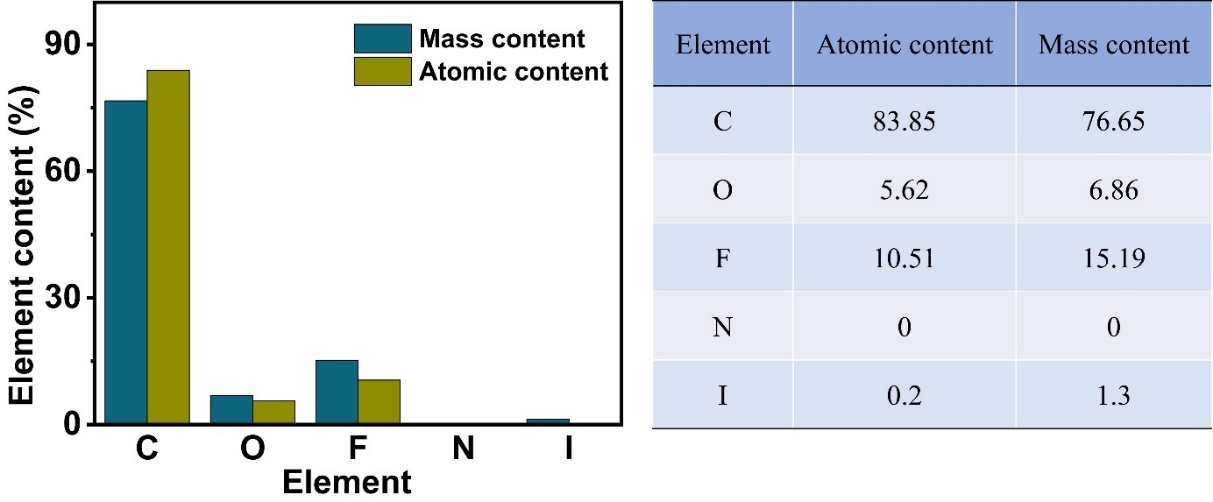
**

**Fig. S12** Atomic and mass content of C, O, F, N and I in R-Gra anode

**
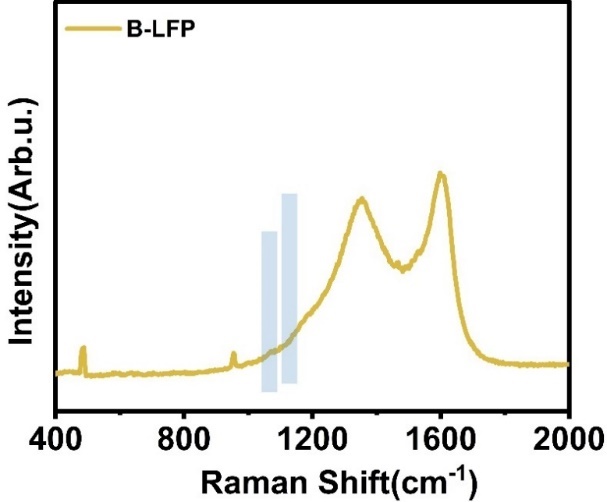
**

**Fig. S13** Raman spectra of B-LFP cathode


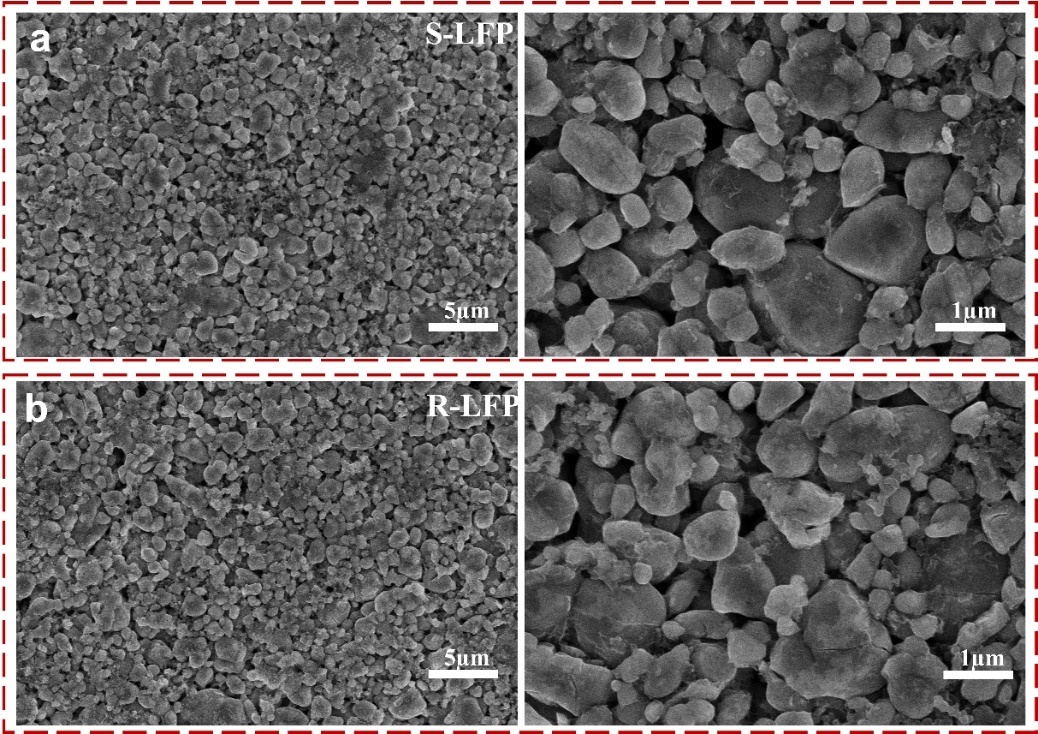


**Fig. S14** SEM images of (**a**) S-LFP and (**b**) R-LFP cathodes


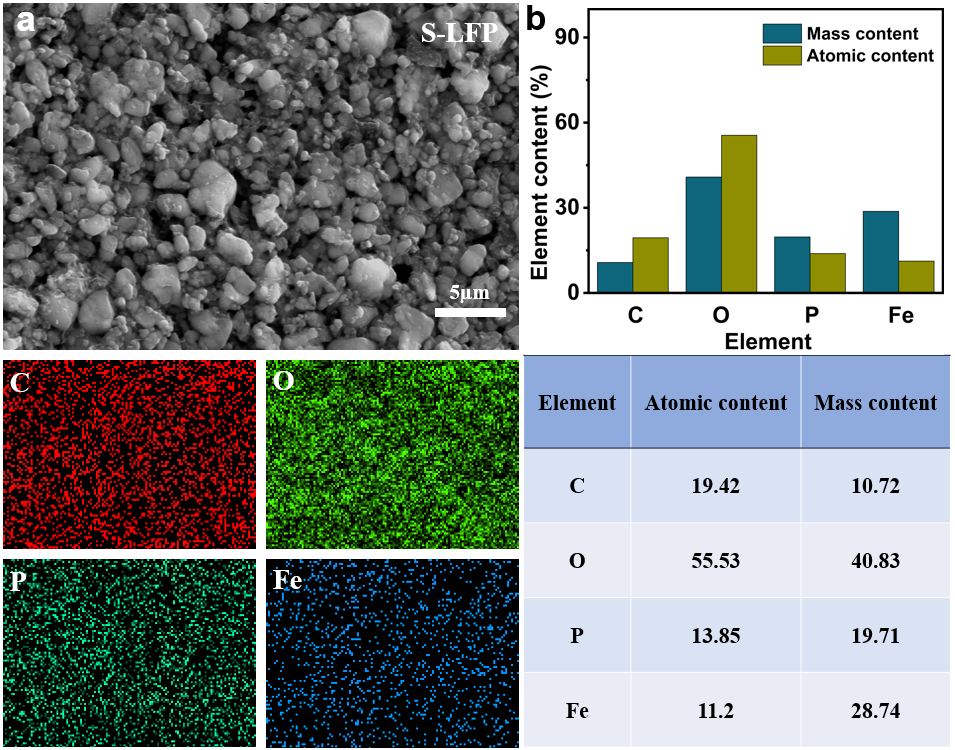


**Fig. S15** (**a**) SEM images and EDS mapping images of S-LFP cathode; (**b**) Atomic and mass content of C, O, P and Fe in S-LFP cathode


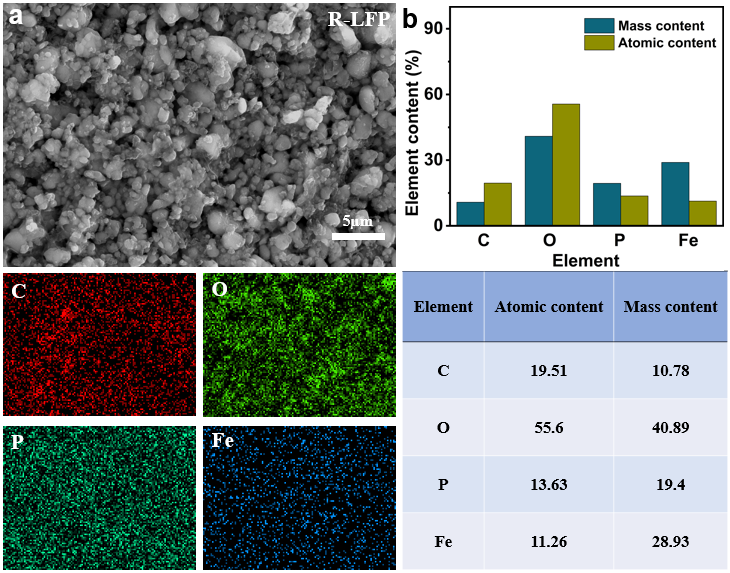


**Fig. S16** (**a**) SEM images and EDS mapping images of R-LFP cathode; (**b**) Atomic and mass content of C, O, P and Fe in R-LFP cathode


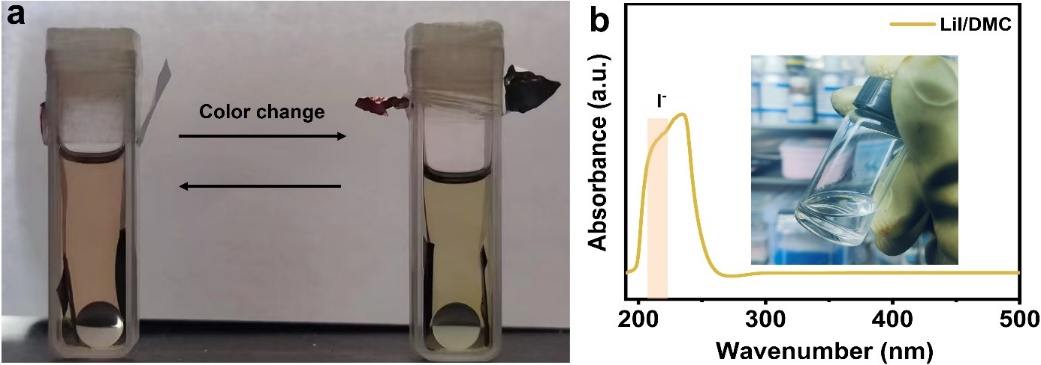


**Fig. S17** (**a**) Color change of the I_2_-DMC solution in homemade-cuvette cells containing the S-LFP and S-Gra electrodes; (**b**) UV-vis spectra and the digital photo of the LiI-DMC solution


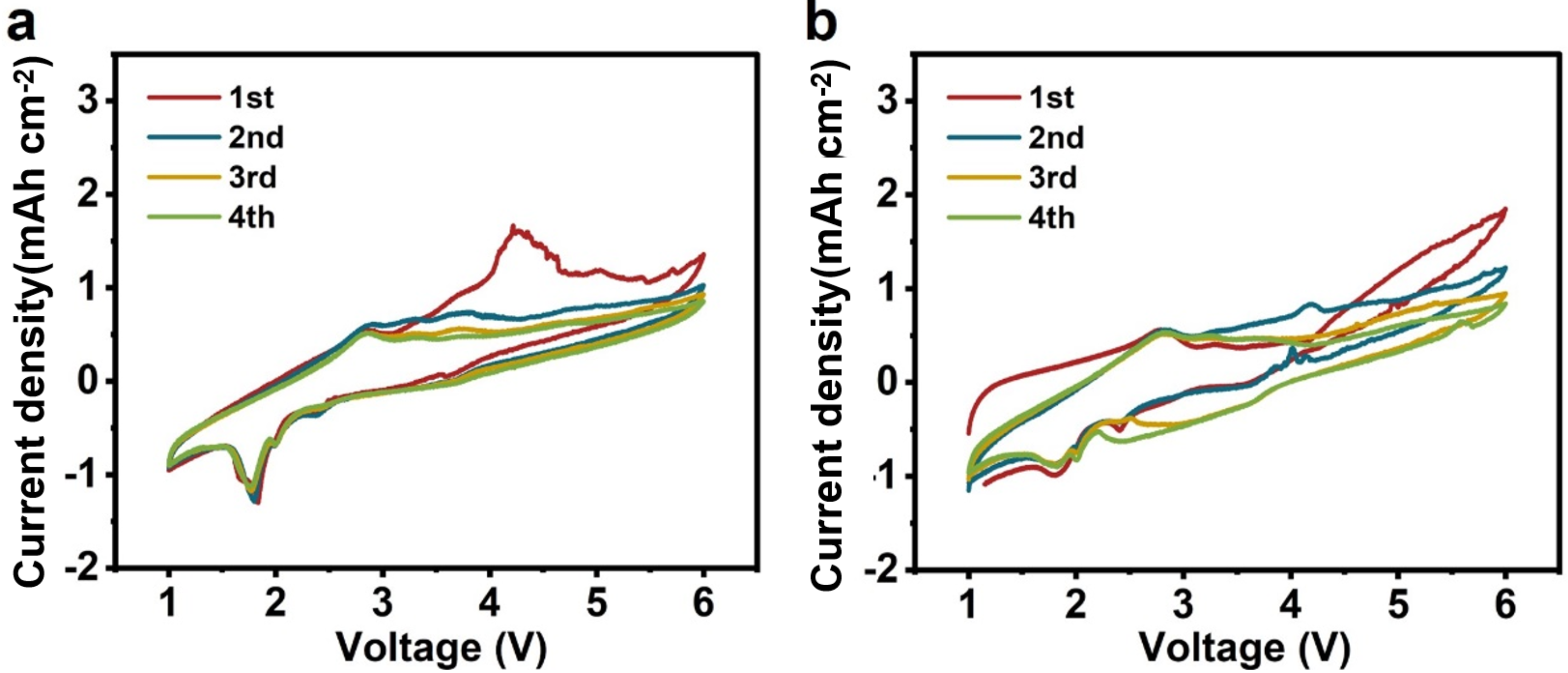


**Fig. S18** CV curves of Li||Al cells in (**a**) LiPF_6_ and (**b**) I_2_-containing electrolytes at 1 mV s^-1^


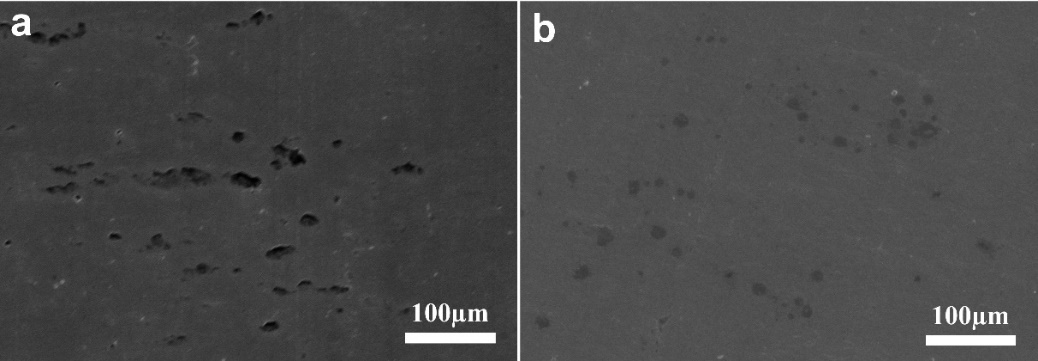


**Fig. S19** SEM images of Al foil in Li||Al cells after CV test in (**a**) LiPF_6_ and (**b**) I_2_-containing electrolytes


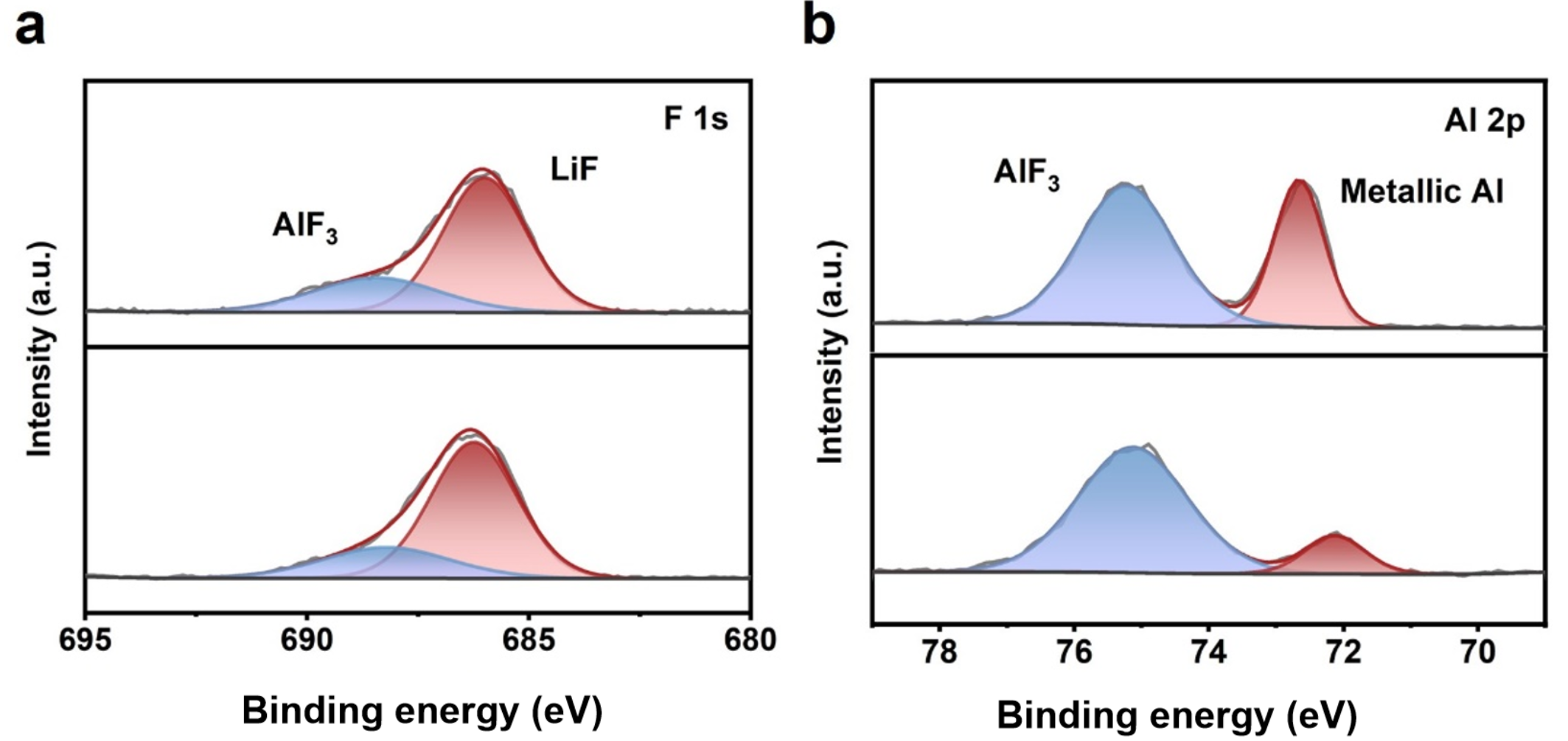


**Fig. S20** High resolution (**a**) F 1s and (**b**) Al 2p spectra of Al foil in Li||Al cells after CV test in LiPF_6_ and I_2_-containing electrolytes


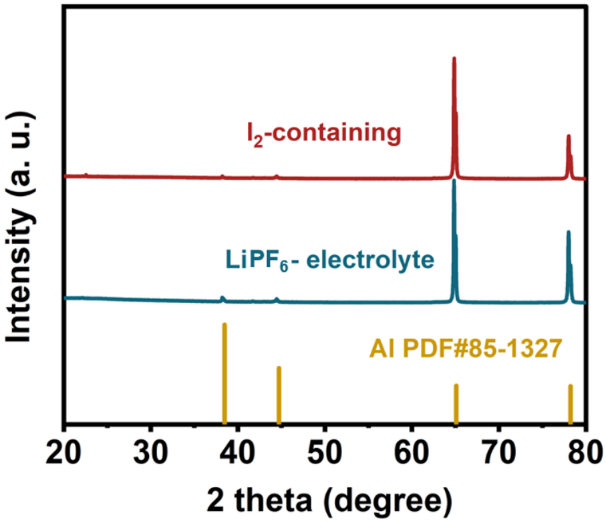


**Fig. S21** XRD patterns of Al foil in Li||Al cells after CV test in LiPF_6_ and I_2_-containing electrolytes


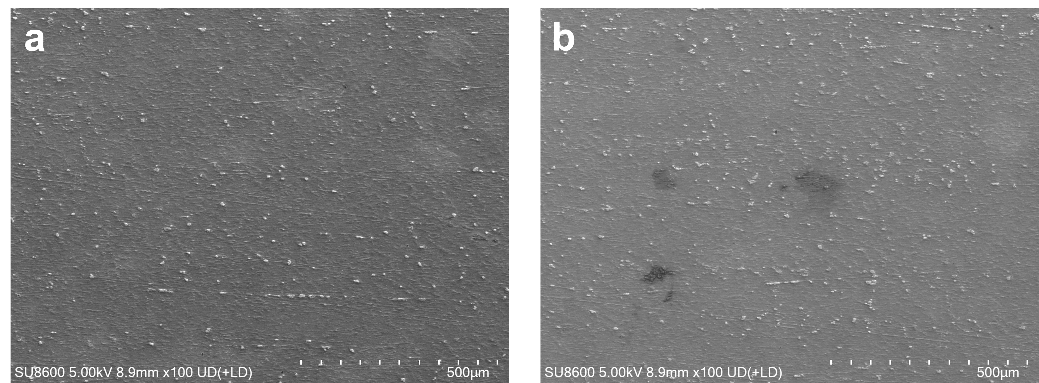


**Fig. S22** SEM images of Cu foil after soaking in (a) LiPF_6_ and (b) I_2_-containing electrolyte for 24 h

**
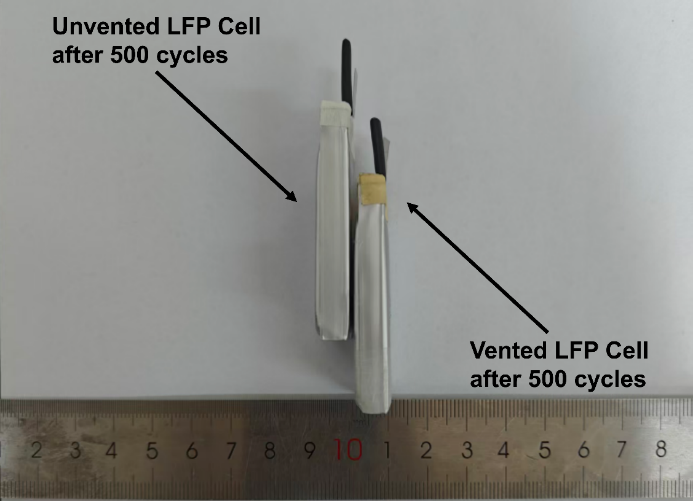
**

**Fig. S23** The appearance of LFP pouch cell without pinhole (left one) and the tape sealed post-injection LFP pouch cell (right one) cycling after 500 cycles


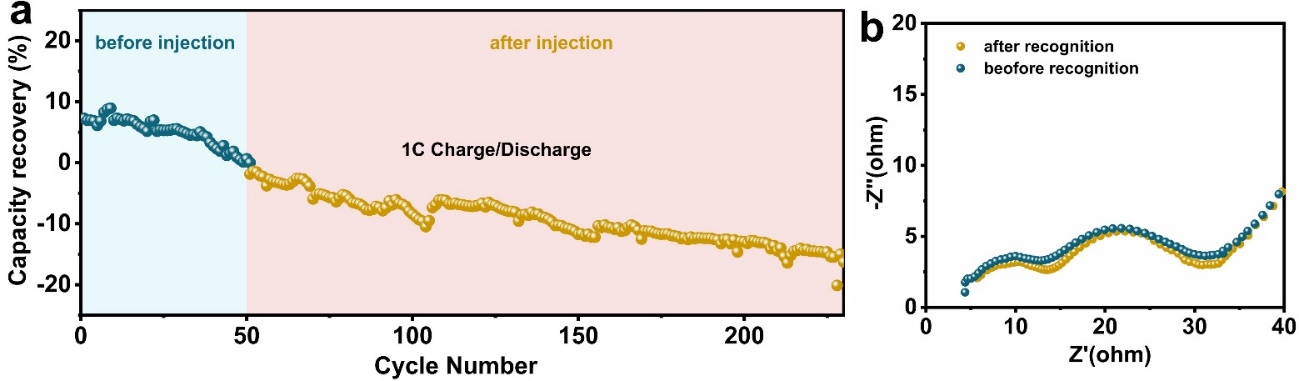


**Fig. S24** (**a**) Capacity recovery ratio and (**b**) EIS spectra of spent pouch cells before and after injecting commercial LiPF_6_ electrolyte without iodine species


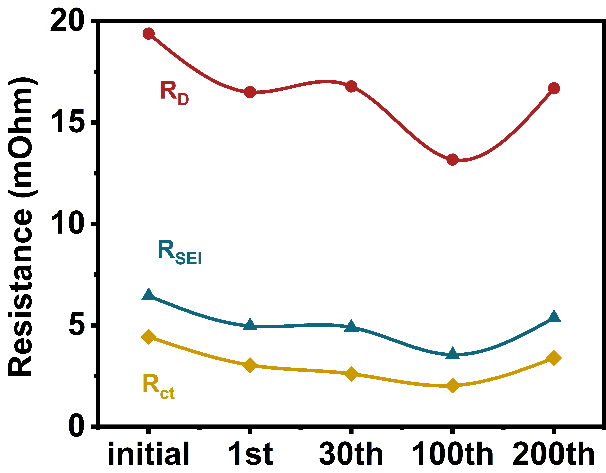


**Fig. S25** Resistance evolution of *R_D_*, *R_ct_* and *R_SEI_*


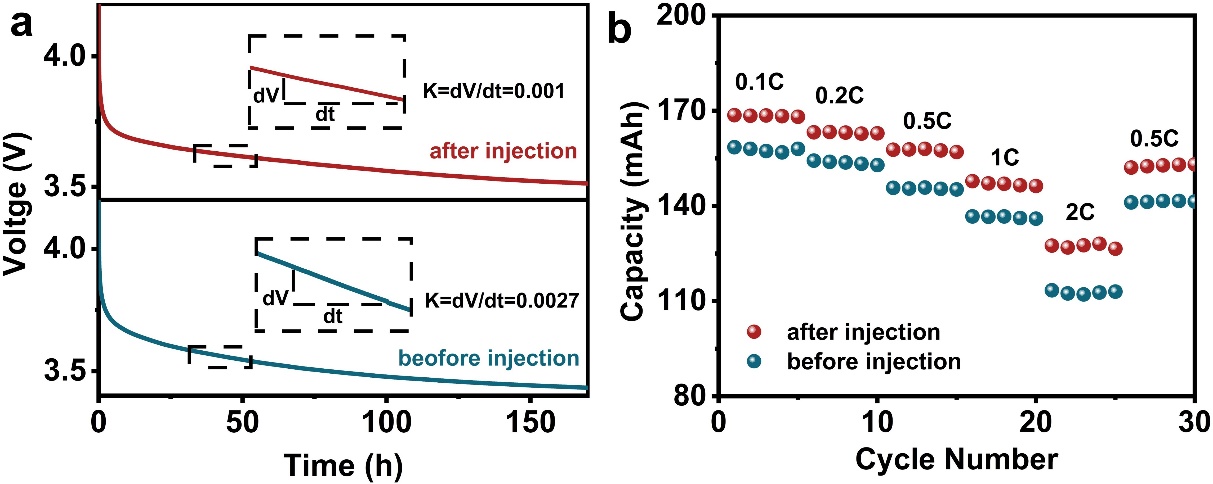


**Fig. S26** (**a**) Self-discharge behavior and (**b**) rate performance of pouch cells before and after injection

**
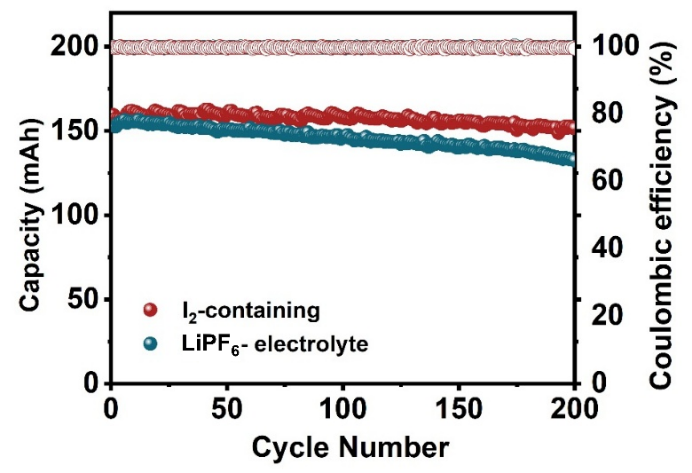
**

**Fig. S27** Cycling performances of LFP pouch cells in raw LiPF_6_ electrolyte and I_2_-containing LiPF_6_ electrolyte


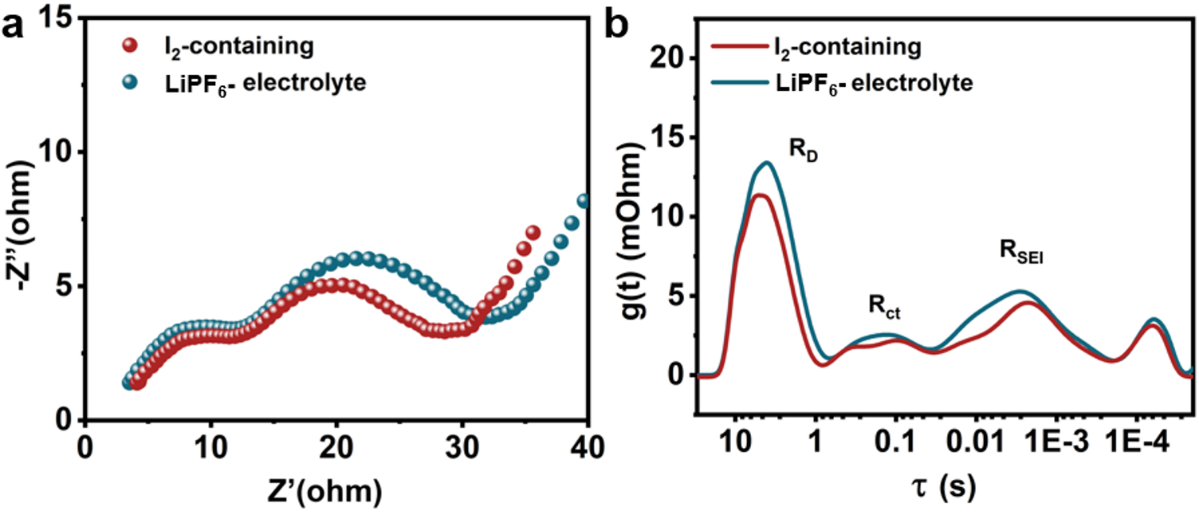


**Fig. S28** (a) EIS spectra and (b) the corresponding DRT spectra of pouch cells in raw LiPF_6_ electrolyte and I_2_-containing LiPF_6_ electrolyte


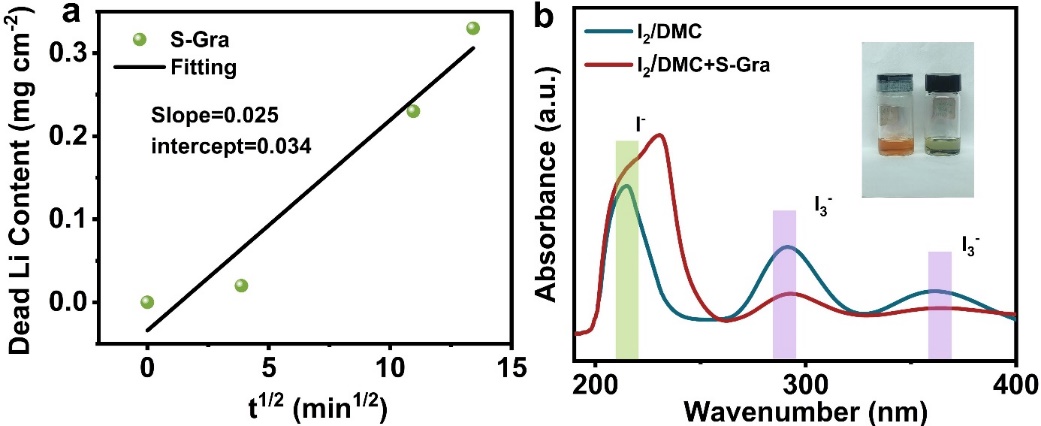


**Fig. S29** (**a**) Variation of dead Li content in S-Gra anode over time and the corresponding linear fitting result; (**b**) UV-vis spectra of raw I_2_-DMC solution and the S-Gra immersed I_2_-DMC solution

**Table S1** Comparison of cycling stability different regeneration techniques cathodes for LiFePO_4_ batteries

| **Regeneration method** | **Cycling performances** | **Capacity retention ratio** | **Refs.** |
| --- | --- | --- | --- |
| **Solid-state regeneration** | after 400 cycles at 1C | 87% | [S1] |
| **Solid-state regeneration** | after 300 cycles at 0.5C | 87.94% | [S2] |
| **Solid-state regeneration** | after 400 cycles at 5C | 88% | [S3] |
| **Solid-state regeneration** | after 300 cycles at 1C | 82% | [S4] |
| **Ex-situ regeneration** | after 350 cycles at 1C | 81.6% | [S5] |
| **Ex-situ regeneration** | after 500 cycles at 2C | 68.1% | [S6] |
| **Chemical relithiation** | after 500 cycles at 0.5C | 90% | [S7] |
| **Electrochemical** | after 300 cycles at 1C | 85.5% | [S8] |
| **Photocatalytic** | after 500 cycles at 1C | 86.63% | [S9] |
| **Injection recovery** | after 300 cycles at 1C | 77.4% | [S10] |
| **Injection recovery** | after 300 cycles at 1C | 91.2% | This work |

**Table S2** Elemental composition content and molar ratios of R-LFP and S-LFP samples based on ICP-OES analysis

|  | **Element content (wt%)** | | | **Molar ratio** | |
| --- | --- | --- | --- | --- | --- |
|  | **Li** | **P** | **Fe** | **Li/P** | **Li/Fe** |
| **S-LFP** | 2.64 | 14.89 | 26.82 | 0.79 | 0.79 |
| **R-LFP** | 3.44 | 14.82 | 26.62 | 1.03 | 1.04 |

**Table S3** Rietveld refinement results of the XRD pattern of S-LFP

| **Phase 1 LiFePO_4_** | | | | | | **Phase 2 FePO_4_** | | | | | | |
| --- | --- | --- | --- | --- | --- | --- | --- | --- | --- | --- | --- | --- |
| **Atom** | **Site** | **Wyckoff positions** | | | **Occupancy** | | **Site** | | **Wyckoff positions** | | | **Occupancy** |
| **Li 1** | **4a** | 0 | 0 | 0 | 0.981 | NA | |  | |  |  |  |
| **Fe 1** | **4c** | 0.2859 | 0.25 | 0.9614 | 0.981 | 4c | | 0.2731 | | 0.25 | 0.930 | 1 |
| **P** | **4c** | 0.0923 | 0.25 | 0.4073 | 1 | 4c | | 0.070 | | 0.25 | 0.397 | 1 |
| **O** | **4c** | 0.1028 | 0.25 | 0.7018 | 1 | 4c | | 0.126 | | 0.25 | 0.636 | 1 |
| **O** | **4c** | 0.4650 | 0.25 | 0.1986 | 1 | 4c | | 0.20 | | 0.25 | -0.19 | 1 |
| **O** | **8d** | 0.1657 | 0.0317 | 0.2515 | 1 | 8d | | 0.155 | | 0.140 | 0.126 | 1 |
| **Li 2** | **4c** | 0.2859 | 0.25 | 0.9614 | 0.019 | NA | |  | |  |  |  |
| **Fe 2** | **4a** | 0 | 0 | 0 | 0.019 | NA | |  | |  |  |  |

**Phase 1 LiFePO_4_:** Space group: Pnma, Rp = 5.57%, Rwp = 4.11%, a= 10.2526, b=5.9460, c=4.6626;

**Phase 2 FePO_4_:** Space group: Pnma, a=9.7434, b= 5.7504, c=4.7142.

**Table S4** Rietveld refinement results of the XRD pattern of R-LFP

| **Phase LiFePO_4_** | | | | | |
| --- | --- | --- | --- | --- | --- |
| **Atom** | **Site** | **Wyckoff positions** | | | **Occupancy** |
| **Li 1** | **4a** | 0 | 0 | 0 | 0.989 |
| **Fe 1** | **4c** | 0.2837 | 0.25 | 0.9748 | 0.989 |
| **P** | **4c** | 0.0950 | 0.25 | 0.4233 | 1 |
| **O** | **4c** | 0.0912 | 0.25 | 0.7486 | 1 |
| **O** | **4c** | 0.4667 | 0.25 | 0.1979 | 1 |
| **O** | **8d** | 0.1649 | 0.0485 | 0.2883 | 1 |
| **Li 2** | **4c** | 0.2837 | 0.25 | 0.9748 | 0.011 |
| **Fe 2** | **4a** | 0 | 0 | 0 | 0.011 |

LiFePO_4_: Space group: Pnma, Rp = 3.97%, Rwp = 3.18%, a= 10.3191 b=6.0019, c=4.6897.

**Table S5** Electrochemical performances of pouch cells at different degraded states before/after injection

|  | | **Capacity**  **(mAh)** | **Capacity recovery**  **(%)** | **Overvoltage**  **(V)** | **DCIR**  **(mΩ)** |
| --- | --- | --- | --- | --- | --- |
| **Cell-A** | **S** | 145.8 | **3.38** | 0.19 | 22.23 |
|  | **R** | **151.19** |  | **0.18** | **21.07** |
| **Cell-B** | **S** | 128.4 | **7.25** | 0.3 | 26.96 |
|  | **R** | **139.86** |  | **0.21** | **23.49** |
| **Cell-C** | **S** | 113.6 | **10.75** | 0.43 | 44.66 |
|  | **R** | **130.8** |  | **0.21** | **24.18** |
| **Cell-D** | **S** | 99.8 | **21.64** | 0.5 | 61.20 |
|  | **R** | **133.99** |  | **0.22** | **23.81** |

The calculation method of capacity recovery ratio (%):

Capacity recovery ratio (%) = (A-B)/C * 100%

Taking the data of Cell-D as an example, A represents the recovered discharge capacity of pouch cells after injection treatment; B is the remaining discharge capacity of spent pouch cells before injection treatment; C is the reversible discharge capacity of this fresh pouch cells without injection treatment. In this table, the values of A, B, C are 133.99, 99.8, and 158 mAh, respectively, *i.e.,* 21.64%=(133.99-99.8)/158*100%.

**Table S6** Quantitative analysis of inactive lithium content in pouch cells at different degraded states

| **Inactive Lithium Content**  **(mg/g)** | | | **Capacity**  **(mAh)** |
| --- | --- | --- | --- |
| **Cell-A** | **S** | **9.013** | 145.8 |
|  | **R** | **/** | **151.19** |
| **Cell-B** | **S** | **12.192** | 128.4 |
|  | **R** | **/** | **139.86** |
| **Cell-C** | **S** | **14.577** | 113.6 |
|  | **R** | **/** | **130.8** |
| **Cell-D** | **S** | **23.484** | 99.8 |
|  | **R** | **/** | **133.99** |

**Table S7** Recycling cost ($/kg cell) comparison for different battery recycling technologies

| Components | Pyro | Hydro | Direct |
| --- | --- | --- | --- |
| Materials | 1.44 | 1.44 | 1.44 |
| Reagent | 0.09 | 0.15 | 0.14 |
| Labor | 0.19 | 0.28 | 0.05 |
| Electricity & Equipment | 0.1 | 0.4 | 0.05 |
| Depreciation | 0.48 | 0.39 | 0 |
| Sewage treatment | 0 | 0.01 | 0 |

**Table S8** Recycling revenue ($/kg cell) comparison for different battery recycling technologies

| Components | Pyro | Hydro | Direct |
| --- | --- | --- | --- |
| Regenerated cell | 0 | 0 | 13.4 |
| Li_2_CO_3_ | 0 | 1.83 | 0 |
| FePO_4_ | 0 | 0.5 | 0 |
| Graphite | 0 | 1.54 | 0 |
| Aluminum | 0 | 0.52 | 0 |
| Copper | 0.56 | 0.56 | 0 |

**Table S9** Recycling profit ($/kg cell) comparison for different battery recycling technologies

| Components | Pyro | Hydro | Direct |
| --- | --- | --- | --- |
| Cost | 2.30 | 2.67 | 1.68 |
| Revenue | 0.56 | 4.95 | 13.4 |
| Profit | -1.74 | 2.28 | 11.72 |

**Table S10** Greenhouse gas (GHG) emissions (kg CO_2_-eq/kg cell) and energy consumption (kJ/kg cell) for different battery recycling technologies

| Components | Pyro | Hydro | Direct |
| --- | --- | --- | --- |
| GHGs | 1.53 | 2.765 | 0 |
| Energy consumption | 18.72 | 16.85 | 0 |

**Supplementary References**

1. M. Xiao, X. Fu, M. Chen, M. Ye, C. Zhu et al., Constructing a homogeneous medium layer to promote the direct regeneration of spent lithium iron phosphate. ACS Appl. Mater. Interfaces **17**(8), 12199–12207 (2025). <https://doi.org/10.1021/acsami.4c20621>
2. L. Song, C. Qi, S. Wang, X. Zhu, T. Zhang et al., Direct regeneration of waste LiFePO_4_ cathode materials with a solid-phase method promoted by activated CNTs. Waste Manag. **157**, 141–148 (2023). <https://doi.org/10.1016/j.wasman.2022.12.002>
3. G. Ji, J. Wang, Z. Liang, K. Jia, J. Ma et al., Direct regeneration of degraded lithium-ion battery cathodes with a multifunctional organic lithium salt. Nat. Commun. **14**(1), 584 (2023). <https://doi.org/10.1038/s41467-023-36197-6>
4. [4] T. Wang, C. Gao, Z. Zheng, W. Yu, M. Wang et al., N-doped carbon layer construction and targeted defect repair enables direct regeneration of spent LiFePO_4_ cathodes. Adv. Funct. Mater. **35**(40), 2502930 (2025). <https://doi.org/10.1002/adfm.202502930>
5. W. Wang, X. Zeng, H. Hu, T. Yang, Z. Ma et al., 1, 2, 3, 4-tetrakis(2-cyanoethoxy)butane (TCEB)-assisted construction of self-repair electrode interface films to improve the performance of 4.5 V pouch LiCoO_2_/artificial graphite full cells operating at 45 ℃. ACS Appl. Mater. Interfaces **13**(50), 59925–59936 (2021). <https://doi.org/10.1021/acsami.1c18252>
6. S. He, S. Huang, X. Liu, X. Zeng, H. Chen et al., Interfacial self-healing polymer electrolytes for Long-Cycle silicon anodes in High-Performance solid-state lithium batteries. J. Colloid Interface Sci. **665**, 299–312 (2024). <https://doi.org/10.1016/j.jcis.2024.03.118>
7. W. Liu, L. Peng, M. Liu, J. Peng, Z. Zeng et al., Versatile chemical repair strategy for direct regeneration of cathode materials from retired lithium-ion battery. Energy Storage Mater. **78**, 104227 (2025). <https://doi.org/10.1016/j.ensm.2025.104227>
8. S. Zhou, J. Du, X. Xiong, L. Liu, J. Wang et al., Direct recovery of scrapped LiFePO_4_ by a green and low-cost electrochemical re-lithiation method. Green Chem. **24**(16), 6278–6286 (2022). <https://doi.org/10.1039/D2GC01640K>
9. X. Lv, J. Lin, X. Sun, Q. Huang, X. Zhang et al., Direct recycling of spent LiFePO_4_ cathodes through photocatalytic correction of anti-site defects. Adv. Mater. **37**(26), e2503398 (2025). <https://doi.org/10.1002/adma.202503398>
10. Y. Gao, H.-M. Zhang, J. Sun, *In-situ* capacity regeneration of degraded lithium-ion batteries using remanufacturing remediator. Energy Storage Mater. **78**, 104248 (2025). <https://doi.org/10.1016/j.ensm.2025.104248>
